# Cysteine Proteases from *V. cundinamarcensis* (*C. candamarcensis*) Inhibit Melanoma Metastasis and Modulate Expression of Proteins Related to Proliferation, Migration and Differentiation

**DOI:** 10.3390/ijms19102846

**Published:** 2018-09-20

**Authors:** Fernanda O. Lemos, Dalton Dittz, Verlane G. Santos, Simone F. Pires, Hélida M. de Andrade, Carlos E. Salas, Miriam T. P. Lopes

**Affiliations:** 1 Department of Pharmacology, Biological Science Institute, Federal University of Minas Gerais-UFMG, Av. Antônio Carlos 6627, Belo Horizonte 31270-901, MG, Brazil; folemos@hotmail.com (F.O.L.); daltondittz@gmail.com (D.D.); verlanegoncalves@gmail.com (V.G.S.); 2 Department of Parasitology, Biological Science Institute, Federal University of Minas Gerais-UFMG, Av. Antônio Carlos 6627, Belo Horizonte 31270-901, MG, Brazil; simonefpires@gmail.com (S.F.P.); helidandrade@gmail.com (H.M.d.A.); 3 Department of Biochemistry and Immunology, Biological Science Institute, Federal University of Minas Gerais-UFMG, Av. Antônio Carlos 6627, Belo Horizonte 31270-901, MG, Brazil; cesbufmg@icb.ufmg.br

**Keywords:** cysteine proteases, antimetastatic, melanoma, cell migration, melanogenesis, cell differentiation

## Abstract

Previous studies showed that P1G10, a proteolytic fraction from *Vasconcellea cundinamarcensis* latex, reduced the tumor mass in animals bearing melanoma, increased in vitro DNA fragmentation and decreased cell adhesion. Here, we present some molecular and cellular events related to the antimetastatic effect induced by the CMS-2 fraction derived from P1G10 in metastatic melanoma B16-F10 and melanocyte Melan-a. Using difference gel electrophoresis and mass spectrometry, we identified four proteins overexpressed in tumor cells, all of them related to proliferation, survival, migration and cell invasion, that had their expression normalized upon treatment with CMS-2: nucleophosmin 1, heat shock protein 65, calcyclin binding protein and eukaryotic translation initiation factor 4H. In addition, some antioxidant and glycolytic enzymes show increased expression after exposure to CMS-2, along with an induction of melanogenesis (differentiation marker). The down regulation of cofilin 1, a protein involved in cell motility, may explain the inhibition of cell migration and dendritic-like outgrowth in B16-F10 and Melan-a, observed after CMS-2 treatment. Taken together, it is argued that CMS-2 modulates the expression of proteins related to metastatic development, driving the cell to a more differentiated-like state. These effects support the CMS-2 antimetastatic activity and place this fraction in the category of anticancer agent.

## 1. Introduction

Our research group has been characterizing the biochemical and pharmacological properties of proteases derived from latex of *Vasconcellea cundinamarcensis*, synonymy *Carica candamarcensis* Hook 1835. *V. cundinamarcensis* is a member of the Caricaceae family common to many areas in South America. The unripe fruit, similar to other members of the genus Carica, contains large amounts of proteolytic enzymes which decrease along with maturation. The ellipsoid fruit, yellow when ripe, contains a thin, aqueous and aromatic pulp, which is consumed cooked to quench the presence of residual proteolytic enzymes [1]. We showed that a cysteine proteinase enriched fraction from *V. cundinamarcensis*, named as P1G10, displays a variety of pharmacological activities, such as fibrinogenolytic, fibrinolytic and antithrombotic activities [2], and tissue repairer effect in cutaneous [3,4,5], and gastric lesions [6,7]. Preclinical studies show that P1G10 is non-toxic when used topically [4] or systemically, with no evidence of mutagenic or genotoxic effects [8]. We recently reported that P1G10 displays a remarkable antitumor activity in a murine melanoma model. The antitumor activity of P1G10 was supported by its pro-apoptotic and anti-angiogenic effect, in addition to its ability for reducing cell adhesion to extracellular matrix (ECM) [9].

These notable biological activities attributed to cysteine proteases from *V. cundinamarcensis* were also observed in other proteolytic enzymes, such as bromelain, papain, and the serine proteases trypsin and chymotrypsin [10,11]. In the case of trypsin and chymotrypsin, their role is controversial as some evidence support the antitumoral effect, while others attribute to them a tumorigenic effect [12,13]. Preclinical and clinical studies showed that plant proteolytic enzymes act as antitumor and chemopreventive agents due to a variety of biological activities, encompassing pro-apoptotic [14,15,16,17] and anti-inflammatory effects [18]. In addition, these enzymes promote the inactivation of endogenous proteases and cytokines [19,20], modulate the expression of several adhesion molecules [21] and tumor suppressor proteins, and inhibit mitogen-activated protein kinases (MAPK)-regulated pathway [18,22,23,24].

Here, we investigated the antimetastatic property of CSM-2, a subfraction containing some of the isoforms found in P1G10 [25], by studying its effects on melanocyte cultured cells and a highly metastatic melanoma cell line. Melanoma is considered an excellent model to study molecular mechanisms of tumorigenesis since these cells are genotypically, phenotypically, and morphologically distinct depending on the disease stage [26]. Using Difference Gel Electrophoresis (DIGE) and Mass Spectrometry (MS) to assess changes in protein expression involved in different signaling cascades, we showed that CMS-2 normalizes the expression of protein related to migration, proliferation and survival in highly murine metastatic melanoma (B16-F10), as well as promoting cell differentiation-like phenotype in B16-F10 and normal murine melanocyte (Melan-a). In these experiments, we show that CMS-2 is a promising antimetastatic agent by modulating molecular and cellular events related to tumorigenesis and metastasis.

## 2. Results

Here, we examined the changes in protein content that may explain the antimetastatic effect of CMS-2, a subfraction obtained from P1G10. Firstly, the fraction P1G10 was separated into three main fractions by CM-Sephadex chromatography, CMS-1 to CMS-3 (Figure S1A), as previously described by Teixeira and coworkers [25]. As expected, CMS-1 and CMS-2 concentrate most of the proteolytic activity, wherein CMS-1 has approximately three-fold specific amidase activity compared to CMS-2 (18.8 and 6.5 nM/min/µg, respectively). Proteins in both fractions show molecular masses of about 23 kDa (Figure S1B).

### 2.1. CMS-2 Fraction, But Not CMS-1, Reduces the Number of Metastatic Points in Lung of Mice Bearing B16-F10 Melanoma

**Figure 1:**
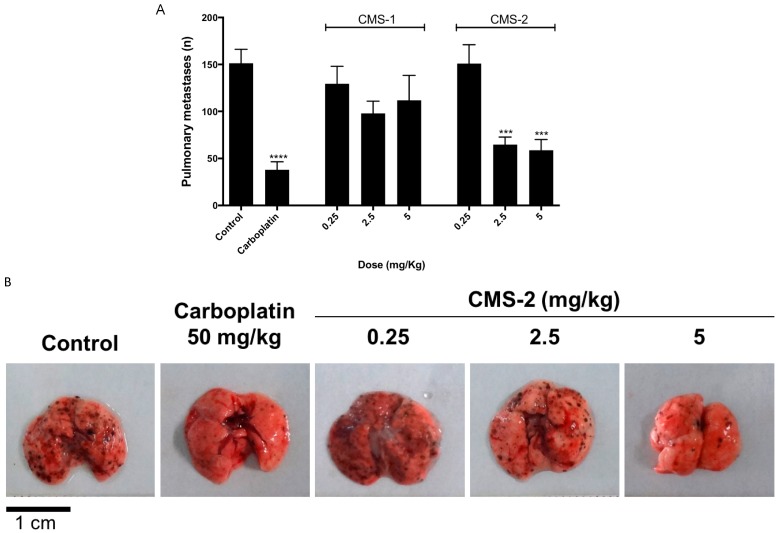
CMS-2 antimetastatic activity in murine melanoma B16-F10. (**A**) The number of metastatic points in lungs of C57/BL6 mice (*n* = 50) after 15 days of subcutaneous treatment with saline (control), carboplatin (positive control), CMS-1 or CMS-2. *** *p* < 0.001 and **** *p* < 0.0001, analysis of variance, Mann–Whitney post-test. (**B**) Representative images of lungs from control or treated mice.

The antimetastatic effect of CMS-1 and CMS-2 was evaluated in a syngeneic model of spontaneous highly metastatic B16-F10 mouse melanoma implanted in C57/BL6 mice. As observed previously, only CMS-2 showed significant antimetastatic activity (Figure 1A,B). At 2.5 and 5 mg/kg, it reduced by ≈50% the number of metastasis compared to saline control. Meanwhile, the number of metastatic points at different doses of CMS-1 were similar to the saline control (Figure 1A).

### 2.2. CMS-2 Fraction Is Cytotoxic to Murine Melanoma and Melanocyte Cell Lines

**Figure 2:**
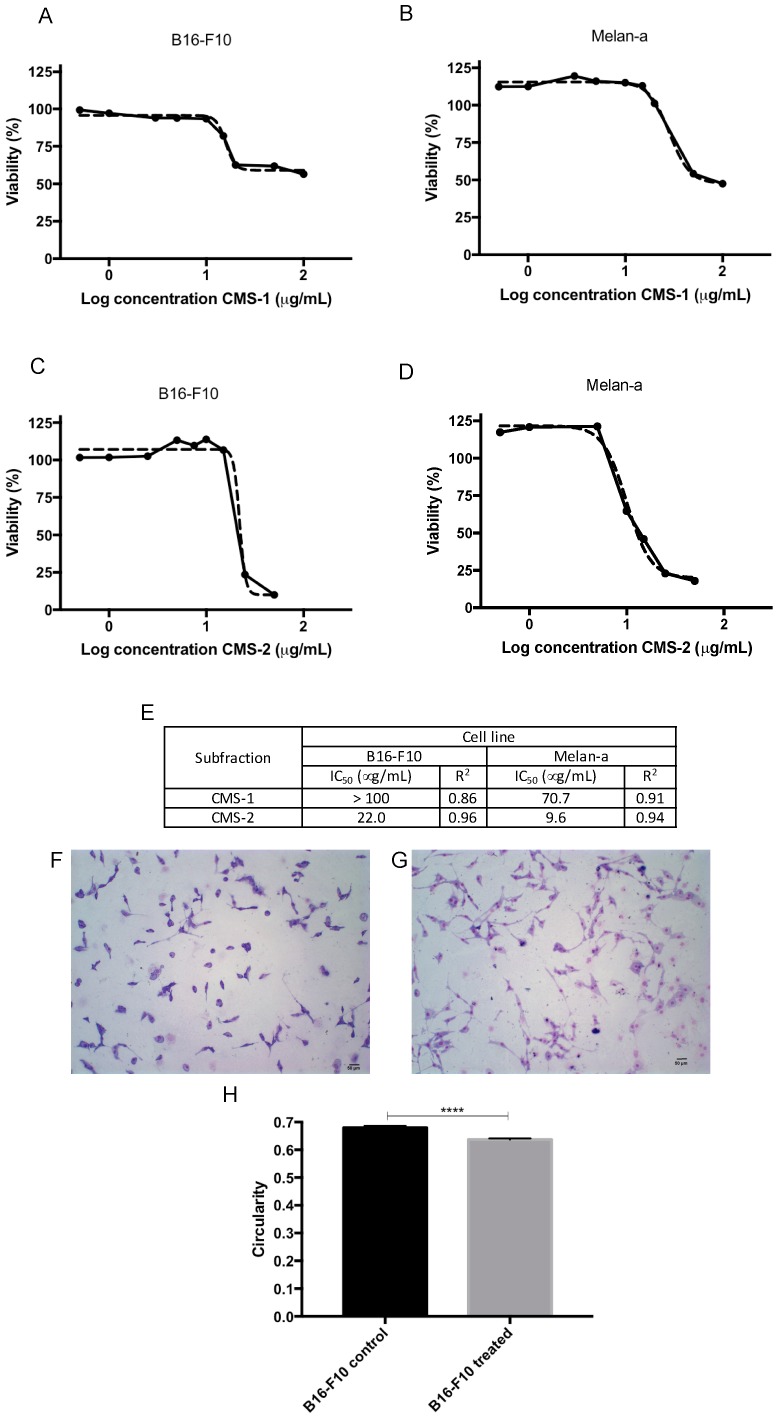
CMS-2, but not CMS-1, treatment reduces cell viability of murine melanoma and melanocyte. Cell viability was evaluated by Resazurin metabolism after 24 h of CMS-1 or CMS-2 exposure (0.5–100 µg/mL) in: B16-F10 (**A**,**C**), respectively; or Melan-a (**B**,**D**), respectively. Absorbance of Resazurin metabolite was spectrophotometrically quantified (570/600 nm) and half maximal inhibitory concentration (IC_50_) was determined through non-linear regression of Log concentration versus absorbance (**E**). (**F**,**G**) Representative images of B16-F10 treated with 10 µg/mL CMS-2 after 24 h. CMS-2 treated cells (**G**) are more circular, quantified in (**H**), fusiform and dendritic compared to the control (**F**). (**H**) Cell circularity was quantified in B16-F10 cells treated with CMS-2 using ImageJ software. **** *p* < 0.001, *t*-test, compared to B16-F10 control.

To verify the cytotoxic effect of CMS-2, the cell viability of B16-F10 and Melan-a cell lines were analyzed by the Resazurin assay. Despite its high proteolytic activity, CMS-1 exhibited low toxicity compared to CMS-2 (Figure 2). The half maximal inhibitory concentration (IC_50_) of CMS-1 was >100 μg/mL in B16-F10 and approximately 71 μg/mL in Melan-a. Meanwhile, CMS-2 remarkably reduced cell viability of melanoma (IC_50_ = 22 μg/mL) and with Melan-a cell line attained IC_50_ = 10 μg/mL. As shown in Figure 2F–H, 10 μg/mL of CMS-2 altered the morphology of B16-F10 (Figure 2G) after 24 h treatment compared to the control B16-F10 (Figure 2F). Under this condition, treated cells were less circular (Figure 2H), more fusiform and displayed more dendrites than the control. No difference was observed in treated Melan-a compared to control Melan-a cells (Figure S2).

### 2.3. The Treatment with CMS-2 Alters the Expression of Proteins Related to Tumorigenesis and Formation of Metastasis 

**Figure 3:**
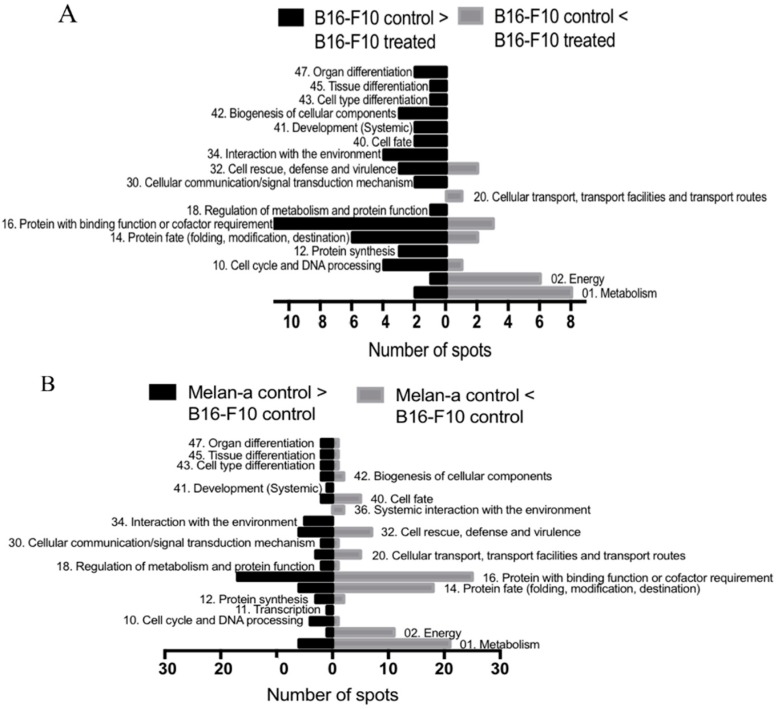

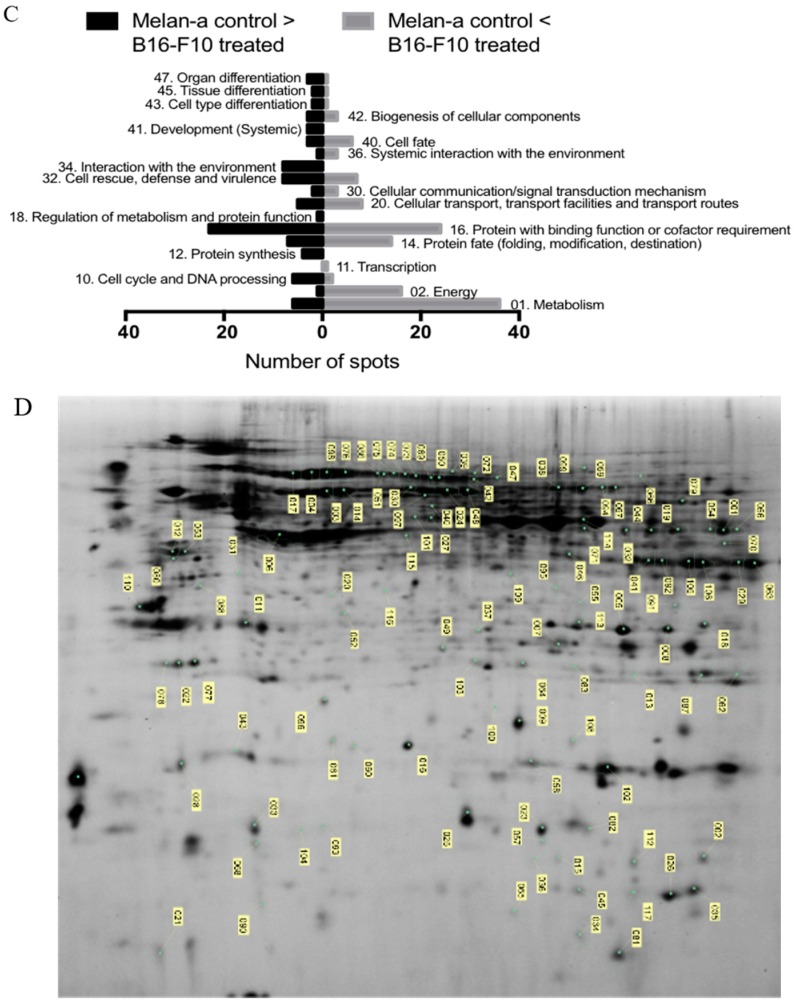
Proteomic analysis of B16-F10 and Melan-a cells treated with CMS-2 including functional assignment of identified proteins: (**A**) comparison between B16-F10 control and treated; (**B**) comparison of Melan-a control to B16-F10 control; and (**C**) comparison between Melan-a control and B16-F10 treated. Exactly 10 µg/mL was added to treated cells. Protein functional classification was based on FunCat (http://mips.helmholtz-muenchen.de/genre/proj/mfungd/index.html) database. (**D**) Image of two-dimensional Difference Gel Electrophoresis (DIGE) (SDS-PAGE 12%, strips IPG 24 cm, pH 3−11 nonlinear) representing an overlay of six gels used to analyzed the protein extracts from B16-F10 control, B16-F10 treated, Melan-a control and Melan-a treated. The spots labeled in this composite image includes all the selected spots for the analysis.

To assess the differential protein expression between B16-F10 and Melan-a (controls) and the corresponding cells treated with 10 μg/mL CMS-2, a two-dimensional DIGE assay was conducted using 24 cm, nonlinear pH 3–11 strips. The representative gel images are shown in Figure S3. After in-gel image analysis by DeCyder 2D software, Version 7.2 (GE Healthcare Bio-Sciences AB, Upsalla, Uppsala County, Sweden), 1128 spots on Gel 1 (B16-F10 and Melan-a controls), 1279 on Gel 2 (Melan-a control and treated), 1409 on Gel 3 (B16-F10 treated and Melan-a control), 1354 on Gel 4 (B16-F10 and Melan-a treated), 1392 on Gel 5 (B16-F10 control and Melan-a treated) and 1444 on Gel 6 (B16-F10 control and treated) were located. Twenty-one spots whose expression was significantly different (*p*-value < 0.05 one-way analysis of variance (ANOVA) and Students’ *t* test) between B16-F10 control and CMS-2 treated B16-F10 cells were selected for MS identification. The comparison between Melan-a control and B16-F10 control yielded 71 spots, while the comparison between Melan-a control and CMS-2 treated B16-F10 cells showed 78 spots. However, the spot pattern was similar when comparing Melan-a control with CMS-2 treated Melan-a cells. From a total of 117 selected spots (Figure 3D), 84 were identified, as summarized in Table S1.

The identified proteins were classified according to FunCat (Functional Catalogue of Proteins) and categorized into 19 diverse functional groups. Figure 3A shows that most protein categories were upregulated (48 proteins) in B16-F10 control compared to CMS-2 treated B16-F10 cells. The exceptions were proteins functionally related to metabolism, energy, cellular transport, transport facilities and transport routes, in which case, a decrease of these proteins was observed following treatment. A comparison of B16-F10 control and Melan-a control showed that most protein categories are more abundant in Melan-a control (Figure 3B). However, proteins involved in metabolism, energy, protein fate (folding, modification, and destination), proteins with a binding role or cofactor requirement (structural or catalytic), cellular transport, transport facilities and routes, cell rescue, defense and virulence, and cell fate had their expression increased in B16-F10 control. Most upregulated proteins in B16-F10 treated with CMS-2 compared to Melan-a control belong to categories of metabolism, energy, transcription, protein fate (folding, modification, and destination), binding function or cofactor requirement (structural or catalytic), cellular transport, transport facilities, transport routes, cellular communication/signal transduction mechanism, systemic interaction with the environment and cell fate (Figure 3C).

**Figure 4:**
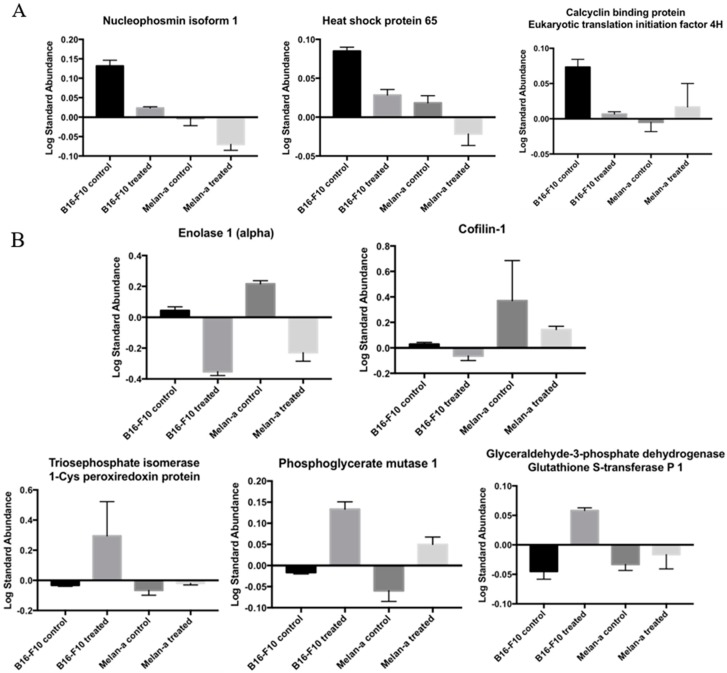
Groups of proteins whose expressions were statistically altered by CMS-2 treatment: (**A**) Group 1 includes proteins whose expression was initially increased in B16-F10 (control) but after CMS-2 treatment normalized their expression (became similar to Melan-a control); and (**B**) Group 2 includes proteins which after CMS-2 treatment altered the expression in both cell lines (similar expression in Melan-a control and B16-F10 control but different expression compared to B16-F10 treated).

Considering the sizable amount of identified proteins, we grouped some of them according to their common profile (Figure 4). The first profile in Figure 4A refers to proteins highly abundant in tumor cells whose levels are depressed upon treatment with CMS-2. *Nucleophosmin* isoform 1 (Spot 12), *heat shock protein 65* (HSP65) (Spot 14), *calcyclin* binding protein and eukaryotic translation initiation factor *4H* (both in Spot 18) belong to this profile. The second group involves proteins whose expression was changed after CMS-2 treatment regardless of the cell type, normal or tumor (Figure 4B). This group includes enolase 1 (α-enolase) (Spot 2) and cofilin-1 (Spot 9), whose abundance decreased after CMS-2 treatment, both in B16-F10 and Melan-a cell lines. Meanwhile, triosephosphate isomerase (Spot 7), 1-Cys peroxiredoxin protein (Spot 7), phosphoglycerate mutase 1 (Spot 8), glyceraldehyde-3-phosphate dehydrogenase (Spot 13) and glutathione S-transferase P 1 (Spot 13) had increased content after CMS-2 treatment (in both cells).

### 2.4. CMS-2 Fraction Inhibits Proliferation of Melanoma and Melanocyte Cell Lines

**Figure 5:**
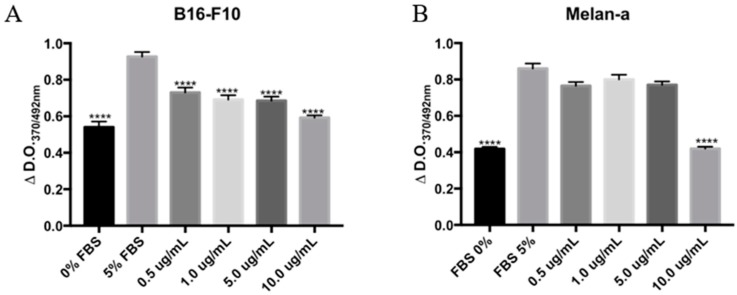
CMS-2 inhibits proliferation of melanoma and melanocyte cell line. Cell proliferation was evaluated by incorporation of bromodeoxyuridine (BrDU) assay after 24 h of CMS-2 treatment (0.5–10 μg/mL). DNA synthesis, represented by the difference in absorbance between 370 and 492 nm for: B16-F10 (**A**); and Melan-a (**B**). **** *p* < 0.001, analysis of variance, Bonferroni post-test compared to 5% fetal bovine serum (FBS) positive control. The negative control was 0% FBS.

The ability of CMS-2 to inhibit DNA synthesis was evaluated by the bromodeoxyuridine (BrdU) assay. As shown in Figure 5, CMS-2 treatment significantly decreased the amount of BrdU incorporated in both B16-F10 and Melan-a cells. CMS-2, at 0.5–10 μg/mL, significantly reduced synthesis of DNA in B16-F10 (Figure 5A) after 24 of treatment. In Melan-a, only at 10 μg/mL CMS-2 inhibited proliferation after 24 h of treatment (Figure 5B). 

### 2.5. CMS-2 Induces Melanogenesis in Melanoma and Melanocyte Cell Lines

**Figure 6:**
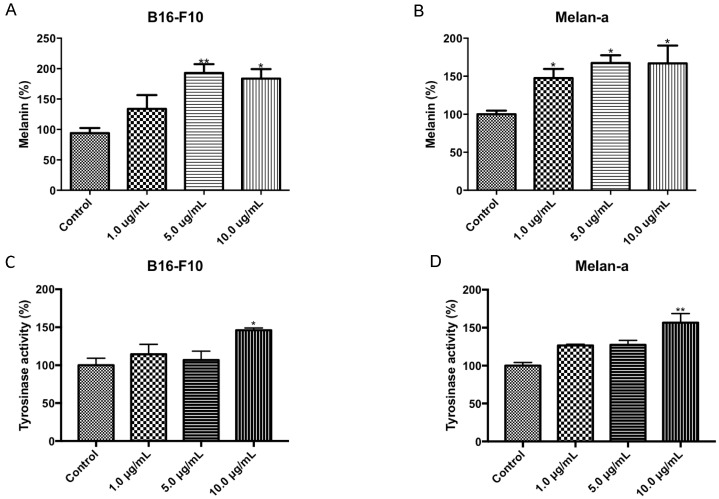
CMS-2 activates melanogenesis in melanoma and melanocyte cell line. Cell melanogenesis was evaluated by measuring the amount of melanin and tyrosinase activity after 24 h of CMS-2 treatment (1–10 μg/mL): melanin content in B16-F10 (**A**) and Melan-a (**B**); and tyrosinase activity in B16-F10 (**C**) and Melan-a (**D**). * *p* < 0.05 and ** *p* < 0.01, analysis of variance, Newman–Keuls post-test.

The effect of CMS-2 on melanogenesis was evaluated by quantification of melanin content and tyrosinase activity (Figure 6). CMS-2 treatment significantly increased the amount of melanin in both cell lines. In B16-F10 at 5 and 10 μg/mL CMS-2, melanin increased by 93% and 84%, respectively, compared to the control (Figure 6A), whereas, in Melan-a, the melanin content increased by 67% at 5 and 10 μg/mL CMS-2 (Figure 6B). Interestingly, tyrosinase, the enzyme responsible for melanin synthesis, showed increased activity in B16-F10 (Figure 6C) and Melan-a (Figure 6D) only at 10 μg/mL CMS-2.

### 2.6. CMS-2 Fraction Inhibits Migration of Melanoma and Melanocyte Cell Lines

**Figure 7:**
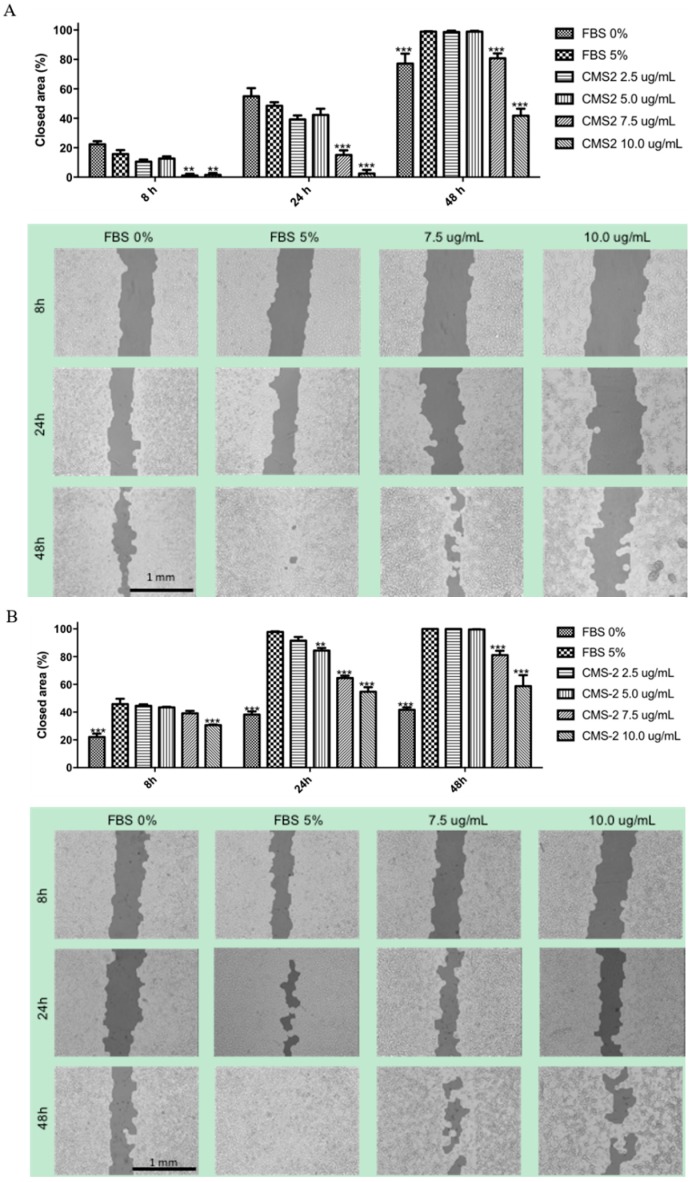
CMS-2 inhibits migration of melanoma and melanocyte cell line. Cell migration was evaluated by the scratch assay 0, 8, 24 and 48 h of CMS-2 treatment (2.5–10 μg/mL). Representative images and quantification of the closed area for: B16-F10 (**A**); and Melan-a (**B**). ** *p* < 0.01 and *** *p* < 0.001, Analysis of variance, Bonferroni post-test. The positive control was 5% fetal bovine serum (FBS) and the negative control was 0% FBS. The images were taken with 4× magnification.

The ability of CMS-2 to reduce or block cell migration was evaluated by the scratch assay. As shown in Figure 7, CMS-2 significantly impaired cell migration both in B16-F10 and Melan-a cells. In B16-F10 and Melan cells (Figure 7A) inhibition of cell migration by CMS-2 was significant at 7.5 and 10 μg/mL during the 48-h interval.

## 3. Discussion

Our group recently showed that the proteolytic fraction P1G10 exerts antitumor activity by reducing tumor mass and increasing survival of mice bearing melanoma. The antitumoral activity of P1G10 was explained by its pro-apoptotic and anti-angiogenic effect, as well as by its ability to reduce cell adhesion to ECM [9]. Further fractionation of the 14 isoforms composing P1G10 was done by CMS-Sephadex chromatography to recover two major proteinase peaks (CMS-1 and CMS-2) and a smaller CMS-3 peak, with CMS-2 containing five of these isoforms [25]. In previous experiments, we showed that CMS-2 is the subfraction responsible for the antitumor and antimetastatic effect of P1G10. Here, by using the experimental metastatic model with rodents, the intention was to evaluate only the colonization capacity of B16-F10 cells replicating early work [27]. The injection of melanoma cells into the tail vein suppresses intermediate stages of tumor development, i.e., migration, invasion of primary tumor cells and modulation of the lymphocyte system. The choice of the experimental metastatic model considered its reproducibility and the economy of time to evaluate the anti-metastatic effect compared to spontaneous metastatic model. Moreover, unpublished results of our research group confirm the antimetastatic activity of CMS2 in B16-F10 melanoma model in which the primary tumor was induced in C57/BL6 mice ear, the “spontaneous metastatic model” [28,29]. In addition, in colon carcinoma metastatic model, 5 mg/kg P1G10 reduces the metastatic points in the liver by 78%, whereas CMS-2 at same concentration reduces the metastatic points by 75%. No antimetastatic effect was observed after similar treatment with CMS-1 [30]. Here, we confirmed that only CMS-2 exerts cytotoxic effect (Figure 2) and reduces points of metastasis in lungs of mice previously injected with B16-F10 murine melanoma cells (Figure 1). However, CMS-1, the most active proteolytical sub-fraction derived from P1G10, did not affect cell viability or metastasis. This evidence suggests that one or more isoforms in CMS-2 exerts the antitumoral action via a specific proteolytic action not found in CMS-1, or through a different activity not yet defined. 

Prior studies demonstrated that two of the cysteine proteases from CMS-2 (CMS2MS2, CMS2MS3) display proliferative activity at nM concentrations, in a fibroblast cell line [31]. Their proliferative effect is independent of proteolytic activity, is partially mediated by Phospholipase C (PLC) activation, and dependent on extracellular signal-regulated kinases (ERK) activation [32]. These results show that CMS2MS2 and CMS2MS3 evoke intracellular signaling cascades independently of a direct proteolytic removal of cell membrane proteins, considered relevant for regulation of growth and cell division [21]. Here, we show that ≈10^−1^ µM doses of CMS-2 reduce proliferation (Figure 5) and cell viability (Figure 2) in normal and tumor cell lines, as observed in Melan-a and B16-F10 cells. To gain insight into the molecular mechanisms accompanying the anti-proliferative and cytotoxic effect of CMS-2, we treated Melan-a and B16-F10 cells with 10 μg/mL of CMS-2 for 24 h and conducted proteomic analysis by DIGE and mass spectrometry, to identify possible changes in protein levels influenced by CMS-2.

DIGE-MS analysis revealed a total of 71 spots displaying different intensities in B16-F10 and Melan-a nontreated groups. Four out of these 71 proteins were over abundant in melanoma, but their levels dropped and became comparable to melanocytic cells after treatment with CMS-2 (Figure 4A). These spots were identified as nucleophosmin isoform 1, HSP65, calcyclin binding protein, and eukaryotic translation initiation factor 4H (eIF4H). Remarkably, all these proteins are related to cell transformation and tumor development. Nucleophosmin-1 is a phosphoprotein found in the nuclei of proliferating cells. This protein has its expression rapidly increased in response to mitogenic stimuli, and increased amounts of the protein are detected in highly proliferating and malignant cells [33]. Nucleophosmin-1 has been implicated in several pathways including mRNA transport, chromatin remodeling, anti-apoptotic cascade and genome stability, and it is commonly overexpressed, mutated, rearranged and sporadically deleted in tumor samples [33,34]. Regarding HSP65, it is well documented that heat shock proteins play a central function in preserving tumor cells survival, by allowing heightened protein synthesis, feeding their proliferative capacity [35]. Heat shock proteins also are ubiquitous molecular chaperones involved in posttranslational folding, stability, activation and maturation of many proteins that are essential mediators of signal transduction and cell cycle progression [35,36]. Yang and colleagues showed that melanoma-bearing mice immunized against HSP65 exhibited slow growth of tumors, decreased pulmonary metastasis points and prolonged survival in line with the data shown here [37]. About calcyclin binding protein, it is highly expressed in several type of cancer, including melanoma [38]. Calcyclin binding protein plays a function in different signaling stages, such as ubiquitination, proliferation, differentiation, tumorigenesis, cytoskeleton dynamic and gene expression [39,40]. Its observed decrease complies with the antitumoral effect showed. Regarding eIFH4, it is known that this protein plays a crucial function in gene expression. Several reports show that dysregulation of many eIFs is associated with malignant transformation and cancer progression as consequence of increased protein synthesis and translation activation of mRNA species that are relevant for cell proliferation and survival [41]. 

It was also observed that some enzymes of the glycolytic pathway (triosephosphate isomerase (TPI), phosphoglycerate mutase 1 (PGM1), and glyceraldehyde-3-phosphate dehydrogenase (GAPDH)) had increased expression in B16-F10 after CMS-2 treatment compared to untreated B16-F10 or Melan-a controls (Figure 3A,C and Figure 4B). Increased expression of aerobic glycolysis is usually observed in actively growing normal and tumoral cells (Warburg effect) [42]. Despite the number of reports describing the Warburg effect, its role in cancer development remains unclear [43]. This is also the case for GAPDH, whose role in cancer prognosis is uncertain. In some cancers, an increase of GAPDH signals tumor progression while in other favors apoptosis [44,45]. A similar situation is described for TPI, its increase in gastric cancers (GC) is linked to a negative prognostic for survival in GC patients [46], while in hepatocellular carcinoma an increased TPI acts as a tumor suppressor [47]. It is likely that yet unknown functions for these enzymes might explain their increases following CMS-2 treatment that support the antitumoral effect observed. 

Alternatively, it is also possible that upregulation of glycolytic enzymes by CMS-2 relates to activation of melanogenesis (Figure 6) as an increase in L-DOPA metabolism favors anaerobic glycolysis (mention below). Activation of melanogenesis leads to expression of hypoxia-induced factor 1α (HIF-1α) and genes whose transcription is targeted by HIF-1α expression, including glycolytic enzymes [48]. Therefore, it is proposed that the increased content of glycolytic enzymes observed could be linked to the increased melanogenesis promoted by CMS-2 in melanoma models. However, as the increase in melanogenesis induced by CMS-2 is seen in both normal and tumor cell lines, this upregulation is not viewed as a tumor-specific effect, even though the melanogenic increase is stronger in B16-F10 cells. 

Remarkably, antioxidant enzymes, such as 1-cys peroxiredoxin and glutathione S-transferase-1, were induced in B16-F10 after CMS-2 exposure compared to B16-F10 and Melan-a controls (Figure 4B). We showed earlier that P1G10, the source of CMS-2 enhanced the contents of thiol groups in gastric cells, thus the induction of glutathione S transferase-1 observed here supports the early result [7]. The melanogenic pathway is upregulated via cAMP by several hormones (MSH, ACTH, and endorphins) including the hormone-like amino acids l-tyrosine and l-DOPA [49,50]. Interestingly, the stimulation of the melanogenic pathway observed in normal and tumor cells (Figure 6) mediated by oxidation of l-tyrosine can be stimulated by NADPH provided by oxidative synthesis of 6-phosphogluconolactone from the pentose pathway as demonstrated in variants of rodent melanoma [51,52]. The upregulation of antioxidant enzymes induced by CMS-2 (1-cys peroxiredoxin and glutathione S-transferase 1) may contribute along with NADPH to dissipate the increased production of damaging ROS intermediates during melanin synthesis. The melanogenic pathway is accompanied by tightly regulated redox reactions involving production of several intermediates, including toxic quinones, semiquinones and reactive oxygen species (ROS) [53,54]. It is unclear if changes in these hormone regulators result from CMS-2 treatment, but an increase in tyrosinase activity, the rate-limiting step enzyme responsible for l-DOPA synthesis was detected when CMS-2 was present at 10 µg/mL. A potential increase in l-DOPA due to the increase in tyrosinase may serve as stimulus shifting the energy metabolism to anaerobic glycolysis, as observed earlier [51]. A possible effect of the Roswell Park Memorial Institute medium (RPMI) on the observed melanogenic effect is ruled out, as the increase in melanogenesis is referred to a control which contains similar tyrosine concentration. The upregulation of antioxidant enzymes by CMS-2 seen here may contribute to dissipate the increased production of damaging ROS intermediates during melanin synthesis.

In addition, CMS-2 promoted downregulation of cofilin-1 and α-enolase in B16-F10 cells compared to B16-F10 or Melan-a controls (Figure 4B). Considering the key function of cofilin-1 during actin polymerization and cell motility [55,56,57], the reduction of this protein explains the inhibitory effect of CMS-2 in cell migration (Figure 7). Cofilin-1 is a small ubiquitous protein (≈19 kDa) that enhances actin action by binding and severing actin filaments during cell migration [57]. Additionally, downregulation of cofilin-1, and consequently stabilization of actin filaments, can explain the morphological changes in B16-F10 observed after 24 h of CMS-2 exposure (Figure 2F–H). The untreated melanocyte and melanoma cells were rounded with epithelial-like shape, while CMS-2 treated cells were less circular and display dendritic-like outgrowths, a characteristic differentiation marker for melanocytes [58]. In the case of α-enolase, also a glycolytic enzyme, its down regulation is seen as a method to reduce the number of lung metastases in immunosuppressed mice injected with pancreatic ductal adenocarcinoma cells (PDAC) a highly aggressive malignancy characterized by rapid progression, invasiveness and resistance to treatment [59].

## 4. Material and Methods

### 4.1. Animals

Male C57/BL6 mice (20–22 g and 7–8-weeks-old) were obtained from the Centro de Bioterismo (CEBIO/ICB/UFMG, Belo Horizonte, Minas Gerais, Brazil). Mice were housed at 22 ± 2 °C under a 12/12 h light/dark cycle with free access to food and water. All animal handling and experimental procedures were conducted with prior approval by the Ethics Committee in Animal Experimentation at the Federal University of Minas Gerais (17 August 2015) and was registered with project identification code # 229/2013, 18 August 2015.

### 4.2. Production of CMS-2

Twenty grams of dried latex from *V. cundinarmarcensis* were dissolved in 100 mL of 1 M sodium acetate containing 25 mM l-cysteine, 5 mM dithiothreitol (DTT) and 10 mM ethylenediaminetetraacetic acid (EDTA) (pH 5.0). This mixture was incubated for 30 min with gentle agitation at room temperature, followed by centrifugation in Sorvall, rotor SS-34 for 10 min at 10,000 *g* and the supernatant filtered through gaze. The filtered solution was chromatographed through a Sephadex G-10 column previously equilibrated with 1 M sodium acetate (pH 5.0). The proteolytic fractions were screened by absorbance at 280 nm and the amidase activity measured by Nα-Benzoyl-dl-arginine 4-nitroanilide hydrochloride (BAPNA) substrate. The proteolytic fraction peak 1 (P1G10) was pooled and incubated with p-chlorine mercury benzoate (pCMB) to reversible inhibit the proteolytic activity. The unreacted pCMB was removed by dialysis against 100 mM acetate buffer, pH 5.0. The resulting solution was chromatographed onto a CM Sephadex C-50 equilibrated and rinsed after sample application with 0.1 M sodium acetate. The protein was eluted with a linear gradient between 0.1 and 1.2 M of sodium acetate, and the protein fractions monitored by absorbance at 280 nm and by amidase activity with BAPNA. The first and second main pool of active protein peaks (CMS-1 and CMS2, respectively) were recovered, concentrated by ultrafiltration (10,000 Da pore size) and stored at −20 °C until use. CMS-2 is composed of five isoforms (CMS2MS1–5) [25].

### 4.3. Cell Culture

The B16-F10 cell line, a murine high-metastatic melanoma, was a gift from the Instituto Ludwig de Pesquisa sobre o Câncer (Sao Paulo, Brazil) and Melan-a cell, developed by Bennet et al. [60], murine melanocyte, was a courtesy of Dr Roger Chammas, Radiology Department, Federal University of São Paulo (Sao Paulo, Brazil). Both cell lines were grown in RPMI medium (GIBCO, Carlsbad, CA, USA) with 1% penicillin/streptomycin (GIBCO, Carlsbad, CA, USA). B16-F10 medium was supplemented with 10% heat-inactivated fetal bovine serum (FBS) (GIBCO, Carlsbad, CA, USA), and Melan-a cells supplemented with 5% FBS and 200 nM of phorbol ester myristate (Sigma, St. Louis, MO, USA). Both cells were cultured at 37 °C in a humidified 5% CO_2_ atmosphere.

### 4.4. In Vivo Metastatic Murine Model

A murine metastatic model was established according to Vantyghem, et al. [61]. Briefly, animals were injected intravenously via tail vein with 100 μL of B16-F10 cells suspension (5 × 10^4^ cells/mouse). Animals were distributed into eight groups (4–12 mice/group): control (saline), three groups given 0.25, 2.5 or 5 mg/kg CMS-1 and three groups receiving similar amounts of CMS-2. Carboplatin (50 mg/kg) was used as a positive control. CMS2 treatment started immediately after tumor cell inoculation and was administered daily by cervical subcutaneous injection. After 15-day treatment, mice were sacrificed in a CO_2_ chamber and the number of metastatic points in lung was visually quantified. No toxicity signs were observed after CMS-1 or CMS-2 at doses above 5 mg/kg.

### 4.5. Cell Viability Assay

The cytotoxic effect of CMS-1 and CMS-2 was evaluated by Resazurin assay [62]. Briefly, exponentially growing cells, seeded in 96-well plates (10^4^ cells/well), were incubated with different concentrations (0.1–100 μg/mL) of sterile CMS-1 or CMS-2 for 24 h. At the end of this period, 10 μL of 10 mg/mL Resazurin (Sigma Chemical Co, St. Louis, MO, USA) was added onto each well and incubated at 37 °C for 2 h. The absorbance of the medium was determined at 570 and 600 nm using the Multiskan GO ELISA reader (Thermo Fisher Scientific, Carlsbad, CA, USA) and the graph was plotted as percent of viability versus CMS-2 concentration. The IC_50_ was determined after calculating the nonlinear regression curve of experimental data.

### 4.6. Proteomic Analysis

#### 4.6.1. Sample Preparation

B16-10 and Melan-a cells were plated on coverslips or 75 cm^2^ flasks (1.5 × 10^6^ cell/flask) and treated with control (PBS) or 10 μg/mL of CMS-2, diluted in culture medium supplemented with 5% FBS, for 24 h. Immediately before cell lysis, images were captured with 4× magnification to quantify the cell circularity using the software Image J (version 2.0.0-rc-54/1.51h; National Institutes of Health, Bethesda, MD, USA and the Laboratory for Optical and Computational Instrumentation, Madison, WI, USA). The cells on coverslips were stained with Giemsa and May Grünwald according to the routine protocol and images were taken in light microscope. The cells were washed 3× with RPMI 1640 medium and incubated with 500 μL of lysis buffer (8 M urea, 2 M thiourea, 4% 3-[(3-cholamidopropyl) dimethylammonio]-1-propanesulfonate (CHAPS), 65 mM DTT, 40 mM Tris base and a protease inhibitor mix (GE Healthcare, South Plainfield, NJ, USA)). After 1 h of incubation with agitation at room temperature, the protein extract was centrifuged at 20,000× *g* for 1 h, and the supernatant was kept at –80 °C until use. Protein concentration was determined using the 2D Quant kit (GE Healthcare, South Plainfield, NJ, USA) according to the manufacturer’s instructions. 

#### 4.6.2. DIGE

To differentially identify the abundance of proteins in B16-F10 control versus treated B16-F10, and Melan-a control versus treated Melan-a, 400 pmol of *N*-hydroxysuccinimidyl-ester derivates of cyanine dyes Cy2, Cy3, and Cy5 (GE Healthcare, South Plainfield, NJ, USA) was used to label 50 μg of each sample, according to the manufacturer. Lysine (1 μL of a 10 mM solution) was added to quench the reaction. An internal standard made of a mix of proteins from each condition (B16-F10 control, B16-F10 treated, Melan-a control and Melan-a treated) was labeled with Cy2. Cy3 and Cy5 were used to label proteins from individual condition. Experiments were performed in 3 replicates and dye-swap was done for all conditions. Cy2, Cy3 and Cy5-labeled proteins were pooled, incubated with 2% DDT, and 2% ampholytes (pH 4−7). The volume was adjusted to 450 μL with sample buffer (7 M urea, 2 M thiourea and 4% CHAPS). IPG strips with 24 cm, pH 3−11 nonlinear (GE Healthcare South Plainfield, NJ, USA) were passively rehydrated and isoelectrofocalized for 90,000 Vh on an Ettan IPGphor system (GE Healthcare Bio-Sciences AB, Upsalla, Uppsala County, Sweden), maximum of 50 μA/strip. The focused strips were equilibrated (50 mM Tris-HCl pH 8.8, 6 M urea, 30% glycerol, 2% SDS, 0.002% bromophenol blue, and 125 mM DTT), following by alkylation with iodoacetamide. The strips were placed onto a 12% SDS-PAGE gel, and electrophoresed in Tris/glycine/SDS buffer at 10 °C and 50 mA/gel.

#### 4.6.3. Image Analysis

Gels were scanned on a Typhoon FLA 9000 unit (GE Healthcare Bio-Sciences AB, Upsalla, Uppsala County, Sweden) with excitation/emission wavelengths specific for Cy2 (488/520 nm), Cy3 (532/580 nm), and Cy5 (633/670 nm). Images were analyzed using DeCyder 2D software, Version 7.0 (GE Healthcare Bio-Sciences AB, Upsalla, Uppsala County, Sweden). After Student’s *t*-test and One-Way ANOVA test, the spots with *p*-value < 0.05 were selected for identification. To manually remove the selected spots, DIGE gels were also stained with colloidal Coomassie Brilliant Blue (CBB) G-250 following procedures described elsewhere [63]. 

#### 4.6.4. Identification of Proteins Matrix-Assisted Laser Desorption Ionization Time-of-Flight/Time-of-Flight Mass Spectrometry (MALDI-TOF/TOF MS)

Selected spots exhibiting differential expression were manually excised, trypsinized, and desalted using Zip-Tips (C18 resin; P10, Millipore Corporation, Bedford, MA, USA) [64]. Approximately 0.5 μL sample solution was mixed with 0.25 μL of saturated matrix solution 10 mg/mL α-cyano-4-hydroxycinnamic acid (Aldrich, Milwaukee, WI, USA) in 50% acetonitrile/0.1% trifluoroacetic acid. Samples were spotted on MTP AnchorChip™ 600/384 (Bruker Daltonics, Bremen, Bremen State, Germany) targets and allowed to dry at room temperature. Raw data for the identification of proteins were obtained on an AB SCIEX MALDI TOF/TOF™ 5800 System (Applied Biosystems, Concord, ON, Canada). Instrument calibration was achieved using a mixture of peptides as a reference (des-Arg1-bradykinin (*m*/*z* = 904,468); angiotensin I (*m*/*z* = 1,296,685); Glu1-fibrinopeptide B (*m*/*z* = 1,570,677) and adrenocorticotropic hormone (18–39) (*m*/*z* = 2,465,199)). Trypsin and keratin contaminating peaks were excluded from the peak lists that were used in the database searching. Each spectrum was produced by accumulating data from 200 consecutive laser shots.

#### 4.6.5. Database Search

MASCOT software (version 2.1, Boston, MA, USA) MS/MS ion search tool (available on http://www.matrixscience.com) was used to search the uninterpreted tandem mass spectra in the National Center for Biotechnology Information (NCBI) database. No restriction of protein molecular weight, one missed trypsin cleavage allowed, nonfixed modifications of methionine (oxidation) and cysteine (carbamidomethylation), pyroglutamate formation at the N-terminal glutamine of peptides with any other post-translational modifications were the parameters applied in the search. For MS spectra, 0.6 kDa was the mass tolerance for the peptides, while for the MS/MS spectra the tolerance was 0.4 kDa. Score values higher than the threshold calculated by MASCOT (*p* < 0.05) were considered as positive to identify a peptide. The proteins were functionally classified using the FunCat–Functional Catalogue of Proteins, MIPS (Munich Information Center for Protein Sequences) (http://mips.helmholtz-muenchen.de/genre/proj/mfungd/index.html) database.

### 4.7. Proliferation Assay

The antiproliferative activity of CMS-2 was evaluated by the incorporation of BrdU assay [65]. Briefly, exponentially growing cells, seeded in 96-well plates (10^3^ for B16-F10 or 10^4^ cells/well for Melan-a), were synchronized (12 h without FBS). Then, the cells were incubated for 24 h with different concentrations (0.5–10 μg/mL) of CMS-2, diluted in culture medium supplemented with 5% FBS, or without FBS as a negative control. Then, 10 μL of BrdU solution was added to each well and incubated for 18 h. At the end of this period, the cells were rinsed and fixed according with the instruction provided by the supplier (Roche^®^, Mannheim, Baden-Württemberg, Germany). The anti-BrdU antibody was applied and the BrdU incorporation was quantified by spectrophotometry at 370 and 492 nm. The results are shown as the Δ of the absorbance. 

### 4.8. Melanogenesis Assay

The melanogenesis activity of CMS-2 was evaluated by quantifying the amount of intracellular melanin [66] and the tyrosinase activity [67]. B16-F10 and Melan-a cells were seeded in 6-well plates (2.0 × 10^5^ cells/well) and then incubated with different concentrations (1–10 μg/mL) of CMS-2 for 24 h. At the end of this period, the monolayer was rinsed with PBS and the cells were lysed. For melanin assay, the cells were lysed with 100 μL of 1 M NaOH and then incubated at 80 °C for 2 h and sonicated for 15 min. The absorbance was determined at 405 nm using the Multiskan GO ELISA reader (Thermo Fisher Scientific, Vantaa, Finland) and the amount of melanin determined using a standard curve of melanin. The graph was plotted as percent of melanin in relation to a control group (100%). For tyrosinase activity assay, the cells were lysed with 1% *v/v* of Triton X-100 and the lysate was centrifuged for 15 min at 10,000 rpm. Forty microliters of the supernatant were mixed with 100 μL of 2 mg/mL L-DOPA solution. The enzymatic reaction was performed at 37 °C for 2 h. The absorbance was determined at 490 nm using the Multiskan GO ELISA reader (Thermo Fisher Scientific, Vantaa, Finland) and the tyrosinase activity was normalized by the protein concentration. The graph was plotted as percent of tyrosinase activity in relation to a control group (100%). 

### 4.9. Migration Assay

The inhibitory effect of CMS-2 on cellular migration was evaluated by the scratch assay [68]. Briefly, exponentially growing cells, plated onto 24-well plates, were allowed to form a confluent monolayer. The cell monolayer was linearly scraped with a p-200 pipet tip. The debris were removed by washing the plate with 1 mL of growth medium. The medium was replaced with 500 μL of CMS-2 (2.5–10 μg/mL), diluted in culture medium supplemented with 5% of FBS. Images of the closure of the scratch were captured at 0, 8, 24 and 48 h after the treatment. The images were quantitatively analyzed using TScratch software (CSE Lab, Zurich, Switzerland). The results are shown as the percent closing area, considering 0% at 0-time interval.

## 5. Conclusions

**Figure 8:**
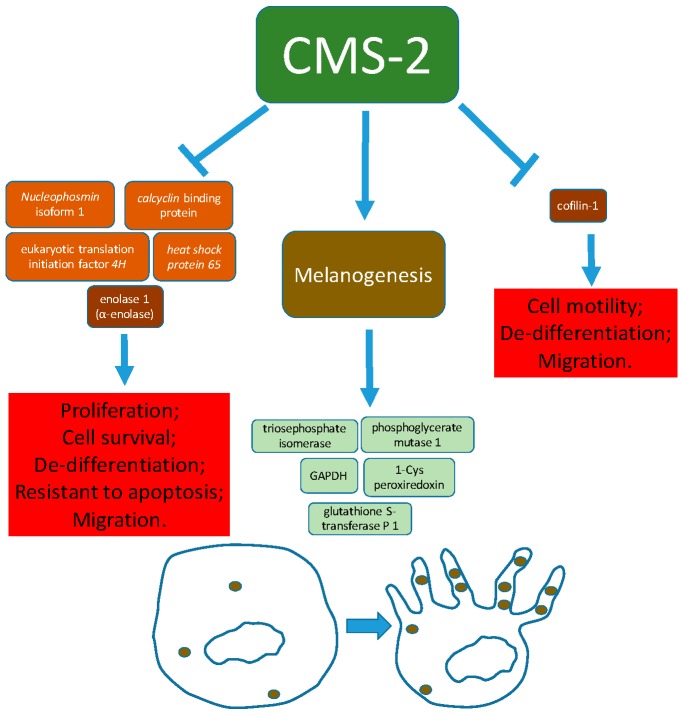
Summary of the molecular and cellular effects observed in melanoma B16-F10 after treatment with CMS-2 fraction from *Vasconcellea cundinamarcensis*.

Here, we show that CMS-2 modulates the expression of proteins related to different cellular events important to metastasis, such as proliferation, survival, motility and dedifferentiation. These variations partially explain the cytotoxicity, inhibition of cell proliferation and migration, and induction of differentiation (melanogenesis and dendrites formation) observed after CMS-2 treatment. A summary with the results obtained is schematized in Figure 8. It is important to highlight that induction of differentiation can be an innovative approach for the development of new antitumor/antimetastatic drugs. Therefore, we demonstrated that CMS-2 is a promising antimetastatic agent, which implies in the necessity of further preclinical studies, especially those focusing on the differentiation cascade.

## Supplementary Materials

Supplementary Materials can be found at http://www.mdpi.com/1422-0067/19/10/2846/s1.
