# Supplementary material for: Cysteine Proteases from V. cundinamarcensis (C. candamarcensis) Inhibit Melanoma Metastasis and Modulate Expression of Proteins Related to Proliferation, Migration and Differentiation
Source: Int J Mol Sci. 2018 Sep 20;19(10):2846. doi: 10.3390/ijms19102846 (PMC6212992; doi:10.3390/ijms19102846)
Supplement: Supplementary file 1 [file ijms-19-02846-s001.zip › ijms-349938-supplementary/5. ijms-349938-supplementary figure.pdf]

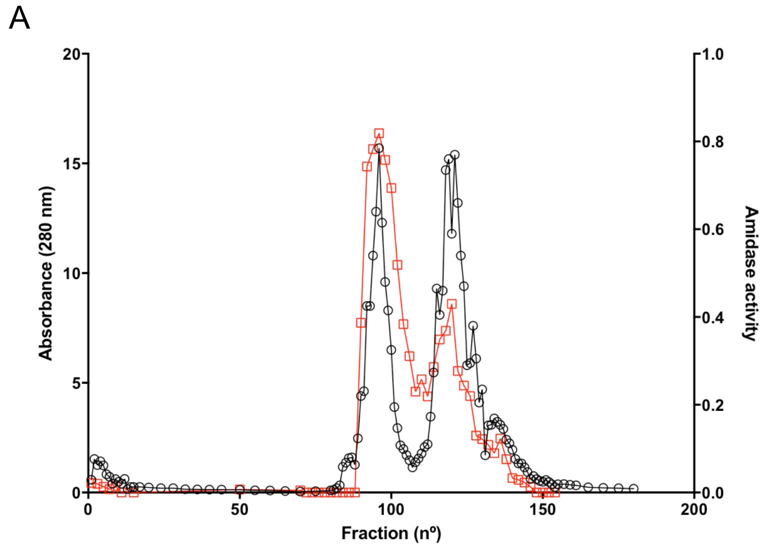

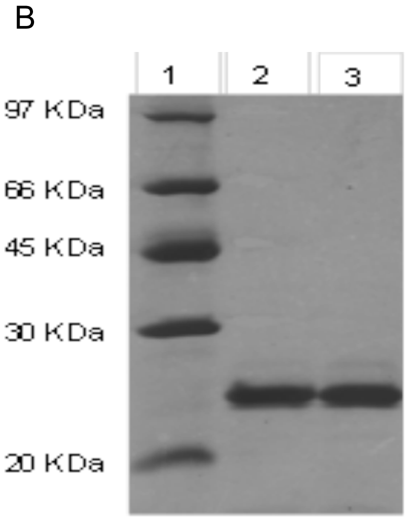

Supplemental figure 1. Biochemistry characterization of proteolytic fractions from *V. cundinamarcensis* latex. A- CM-Sephadex column was equilibrated with sodium acetate 0.1 M (pH 5.0) before injection of P1G10, a proteolytic fraction obtained from *V. cundinamarcensis*, followed by elution with a linear sodium acetate gradient (from 0.1 to 1.2M). Absorbance at 280 nm (black curve) and amidase activity (red curve) were determined for each fraction. Two main fractions were collected: CMS-1 and CMS-2. B- SDS-PAGE electrophoresis profile of CMS-1 and CMS-2. Thirty micrograms of CMS-1 (lane 2) or CMS-2 (lane 3) were resolved by SDS-PAGE and stained with Coomassie blue. Lane 1, molecular mass ladder.

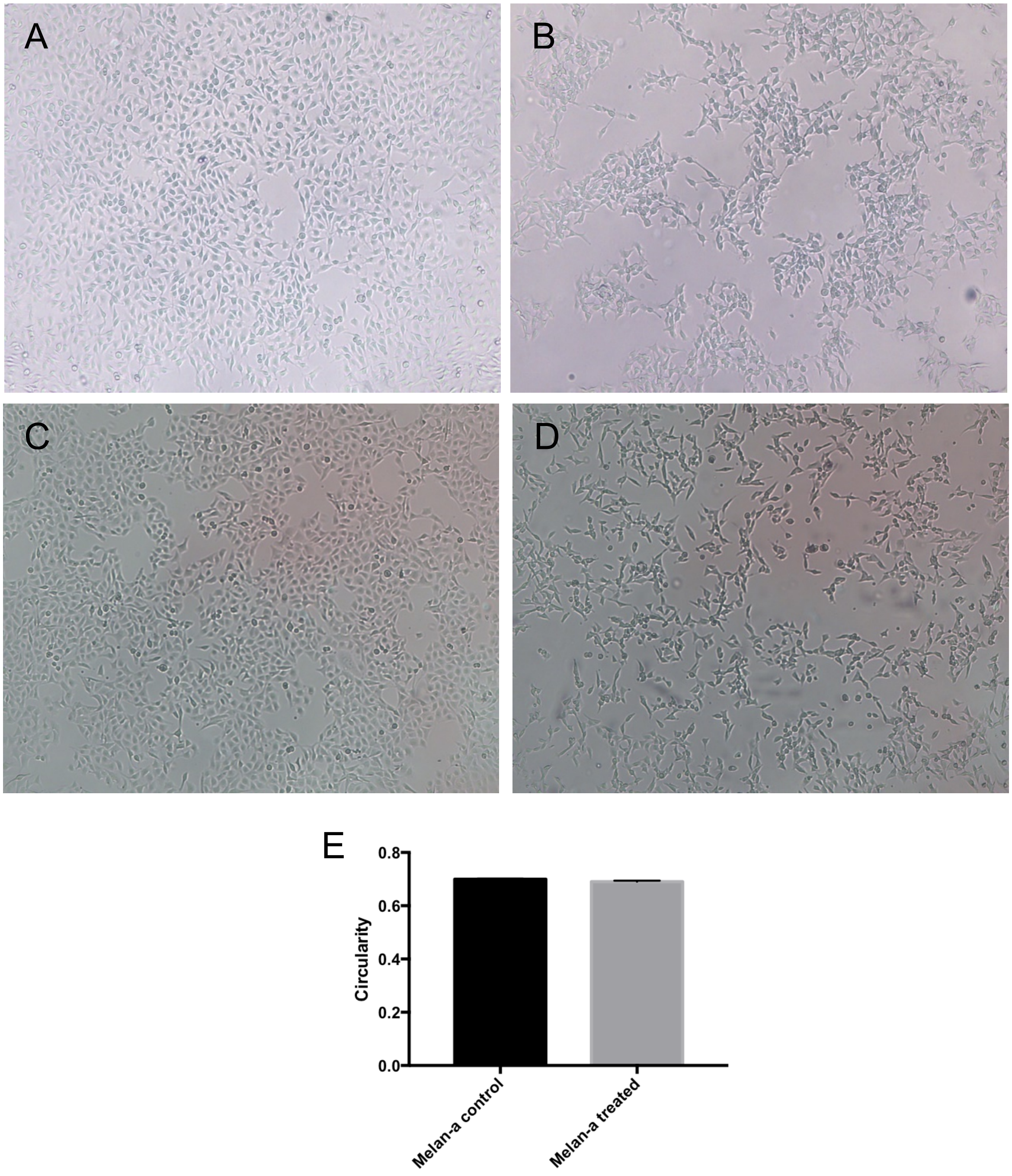

Supplemental figure 2. Representative images of B16-F10 and Melan-a after 24 h of 10 µg/mL CMS-2 treatment. B16-F10 treated cells are more fusiform and dendritic compared to the control. A: control B16-F10; C: control Melan-a; B: treated B16-F10; and D: treated Melan-a. 4X magnification. (E) Cell circularity was not different Melan-a cells treated with CMS-2 compared to control.

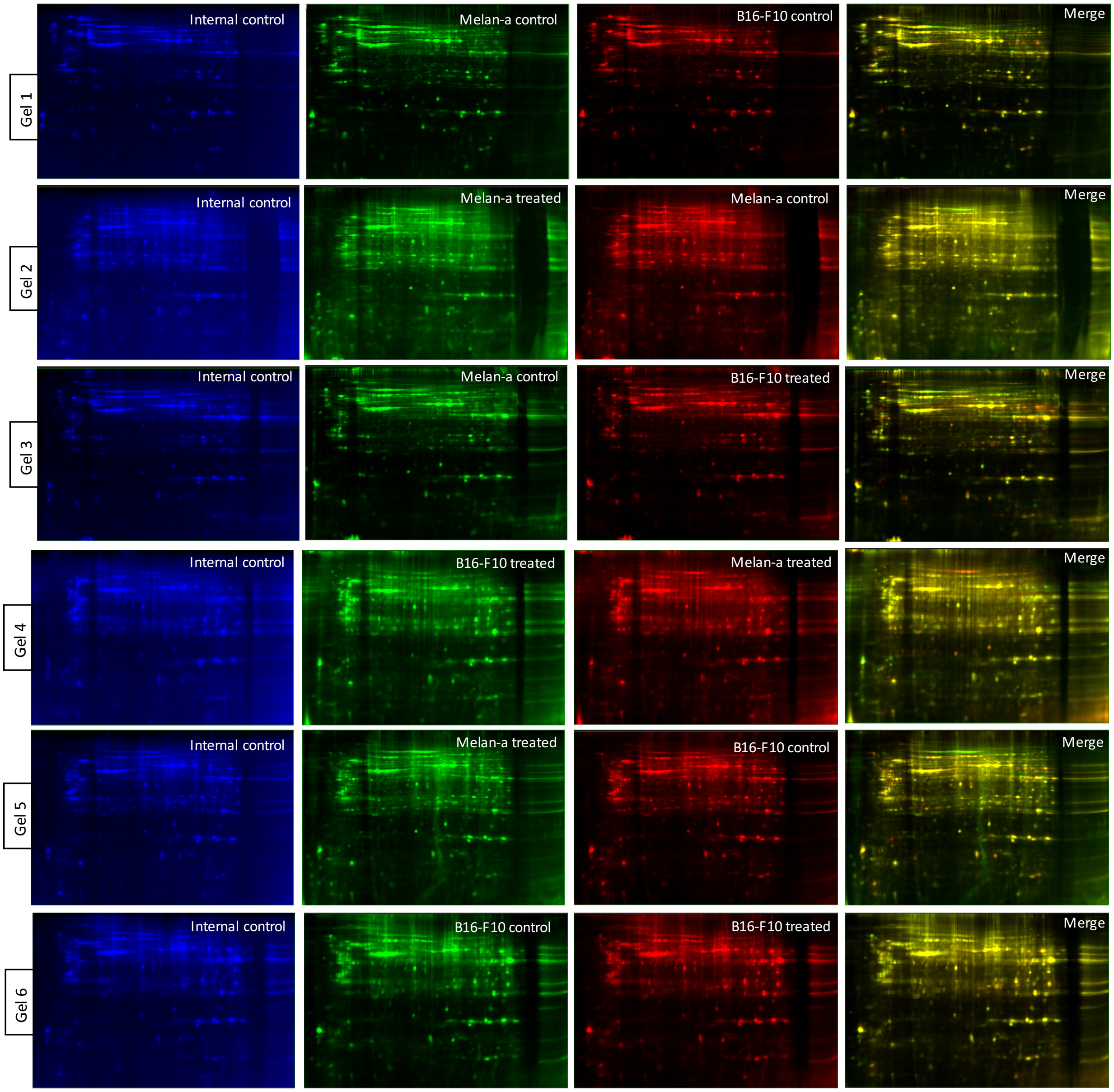

Supplemental figure 3. 2-D DIGE images of cell lysate from B16-F10 and Melan-a controls or treated with CMS-2. Blue: internal control labeled with Cy2 and composed by a mixture of the protein extracts from each conditions (B16-F10 control, B16-F10 treated, Melan-a control and Melan-a treated); Green and red: protein extract from each condition labeled with Cy3 (green) or Cy5 (red). Merge: overlap of the Cy2, Cy3 and Cy5 labeled images.
