# Supplementary material for: Cysteine Proteases from V. cundinamarcensis (C. candamarcensis) Inhibit Melanoma Metastasis and Modulate Expression of Proteins Related to Proliferation, Migration and Differentiation
Source: Int J Mol Sci. 2018 Sep 20;19(10):2846. doi: 10.3390/ijms19102846 (PMC6212992; doi:10.3390/ijms19102846)
Supplement: Supplementary file 1 [file ijms-19-02846-s001.zip › ijms-349938-supplementary/5. ijms-349938-supplementary table.pdf]

Supplemental table 1: Proteins differently expressed among comparison of B16-F10 control, B16-F10 treated, Melan-a control and Melan-a treated

Spot	Protein	GI	Mr/pI	Score	Coverage (%)	Peptides	Abundance
1	phosphoglycerate kinase	gi|202423	/44508<br>/7.53	658	22	K.LGDVYVNDAFGTAHR.A<br>K.ALESPERPFLAILGGAK.V<br>K.ITLPVDFVTADKFDENAK.T<br>K.QIVWNGPVGVFEWEAFAR.G<br>K.QIVWNGPVGVFEWEAFAR.G<br>K.DCVGPEVENACANPAAGTVILLENLR.F	BC<BT<br>1.26<br>MC<BC<br>2.33<br>MC<BT<br>2.92
2	Enolase 1, (alpha)	gi|59808815	/47128<br>/6.16	667	23	R.IGAEVYHNLK.N<br>R.GNPTVEVDLYTAK.G<br>R.YITPDQLADLYK.S<br>K.LAQSNGWGVMVSHR.S<br>R.AAVPSGASTGIYEALELR.D<br>K.LAMQEFMILPVGASSFR.E<br>K.AGYTDQVVIGMDVAASEFYR.S	BC>BT<br>2.61<br>MC=BC<br>MC>BT<br>3.54
3	heat shock protein 65	gi|51455	/60903<br>/5.91	871	16	R.TVIIEQSWGSPK.V<br>R.AAVEEGIVLGGGCALLR.C<br>K.ISSVQSIVPALEIANAHR.K<br>R.KPLVIIAEDVDGEALSTLVLNR.L<br>K.LVQDVANNTNEEAGDGTTTATVLAR.S	BC>BT<br>1.14<br>MC=BC<br>MC=BT
	put. beta-actin (aa 27-375)	gi|49868	/39161<br>/5.78	629	22	R.AVFPSIVGR.S<br>R.GYSFTTTAER.E<br>K.QEYDESGPSIVHR.K<br>K.IWHHTFYNELR.V<br>K.SYELPDGQVITIGNER.F<br>K.DLYANTVLSGGTTMYPGIADR.M<br>K.DLYANTVLSGGTTMYPGIADR.M	
4	heat shock protein 70 cognate	gi|309319	70793<br>5.37	646	14	K.FELTGIPPAPR.G<br>K.VEIIANDQGNR.T<br>R.FEELNADLFR.G<br>R.RFDDAVVQSDMK.H<br>R.TTPSYVAFTDTER.L<br>K.STAGDTHLGGEDFDNR.M<br>K.TVTNAVVTVPAYFNDSQR.Q	BC>BT<br>1.34<br>MC=BC<br>MC=BT

5	phosphoglycerate mutase 1	gi|114326546	28814<br>6.67	824	54	R.VLIAAHGNSLR.G<br>R.HGESAWNLENR.F<br>R.DAGYEFDICFTSVQK.R<br>R.FSGWYDADLSPAGHEEAK.R<br>K.NLKPIKPMQFLGDEETVR.K<br>K.NLKPIKPMQFLGDEETVR.K<br>R.SYDVPPPPMEPDHPFYSNISK.D<br>R.SYDVPPPPMEPDHPFYSNISK.D	BC<BT<br>1.22<br>MC<BC<br>1.27<br>MC<BT<br>1.55
6	gamma-actin	gi|809561	40992<br>5.56	691	25	K.AGFAGDDAPR.A<br>R.GYSFTTTAER.E<br>R.AVFPSIVGRPR.H<br>K.QEYDESGPSIVHR.K<br>K.IWHHTFYNELR.V<br>K.SYELPDGQVITIGNER.F<br>K.DLYANTVLSGGTTMYPGIADR.M<br>K.DLYANTVLSGGTTMYPGIADR.M	BC>BT<br>1.13<br>MC=BC<br>MC=BT
	put. beta-actin (aa 27-375)	gi|49868	39161<br>5.78	629	22	R.AVFPSIVGR.S<br>R.GYSFTTTAER.E<br>K.QEYDESGPSIVHR.K<br>K.IWHHTFYNELR.V<br>K.SYELPDGQVITIGNER.F<br>K.DLYANTVLSGGTTMYPGIADR.M<br>K.DLYANTVLSGGTTMYPGIADR.M	
7	triosephosphate isomerase	gi|54855	26679<br>6.90	516	25	K.IAVAAQNCYK.V<br>K.SNVNDGVAQSTR.I<br>R.HVFGESDELIGQK.V<br>K.TATPQQAQEVHEK.L<br>K.DLGATWVVLGHSER.R<br>R.RHVFGESDELIGQK.V	BC<BT<br>1.42<br>MC=BC<br>MC<BT<br>1.56
	1-Cys peroxiredoxin protein	gi|4139186	24825<br>5.98	94	8	R.NFDEILR.V<br>K.LSILYPATTGR.N	

8	phosphoglycerate mutase 1	gi|114326546	28814<br>6.67	824	54	R.HYGGLTGLNK.A<br>R.VLIAAHGNSLR.G<br>R.HGESAWNLENR.F<br>R.ALPFWNEEIVPQIK.E<br>R.DAGYEFDICFTSVQK.R<br>R.FSGWYDADLSPAGHEEAK.R<br>K.NLKPIKPMQFLGDEETVR.K<br>K.NLKPIKPMQFLGDEETVR.K<br>R.YADLTEDQLPSCESLKDTIAR.A	BC<BT<br>1.25<br>MC=BC<br>MC<BT<br>1.37
9	cofilin-1	gi|6680924	18548<br>8.22	519	40	K.MLPDKDCR.Y<br>K.AVLFCLSEDKK.N<br>K.LGGSAVISLEGKPL.-<br>K.HELQANCYEEVK.D<br>K.HELQANCYEEVKDR.C<br>K.EILVGDVGQTVDDPYTTFVK.M	BC>BT<br>1.35<br>MC=BC<br>MC>BT<br>1.50
10	pyruvate kinase M	gi|551295	57824<br>7.58	687	24	K.GDYPLEAVR.M<br>R.LDIDSAPITAR.N<br>R.NTGIICTIGPASR.S<br>K.IYVDDGLISLQVK.E<br>K.DAVLNAWAEDVDLR.V<br>R.FDEILEASDGIMVAR.G<br>R.LNFSHGTHEYHAETIK.N<br>R.EAEAAIYHLQLFEELR.R<br>R.EATESFASDPILYRPVAVALDTK.G	BC<BT<br>1.12<br>MC=BC<br>MC=BT
11	heat shock protein 70 cognate	gi|309319	70793<br>5.37	646	14	K.FELTGIPPAPR.G<br>K.VEIIANDQGNR.T<br>R.FEELNADLFR.G<br>R.RFDDAVVQSDMK.H<br>R.TTPSYVAFTDTER.L<br>K.STAGDTHLGGEDFDNR.M<br>K.TVTNAVVTVPAYFNDSQR.Q	BC>BT<br>1.12<br>MC>BC<br>1.16<br>MC>BT<br>1.30

	Rho GDP dissociation inhibitor (GDI) alpha	gi|13435747	23377<br>5.12	566	26	K.YIQHTYR.K<br>K.TDYMVGSYGPR.A<br>K.IDKTDYMVGSYGPR.A<br>R.VAVSADPNVPNVIVTR.L<br>K.SIQEIQELDKDDESLR.K<br>K.SIQEIQELDKDDESLRK.Y	
12	nucleophosmin isoform 1	gi|6679108	32540<br>4.62	417	19	K.DLKPSTPR.S<br>K.VDNDENEHQLSLR.T<br>R.MTDQEAIQDLWQWR.K<br>R.MTDQEAIQDLWQWR.K<br>K.MSVQPTVSLGGFEITPPVVLR.L<br>K.MSVQPTVSLGGFEITPPVVLR.L	BC>BT<br>1.30<br>MC<BC<br>1.40<br>MC=BT
13	glyceraldehyde-3-phosphate dehydrogenase	gi|6679937	35787<br>8.44	442	19	R.VPTPNVSVVDLTCR.L<br>K.LVINGKPITIFQER.D<br>K.LISWYDNEYGYSNR.V<br>K.VIHDNFGIVEGLMTTVHAITATQK.T	BC<BT<br>1.26<br>MC=BC<br>MC<BT<br>1.23
	glutathione S-transferase P 1	gi|10092608	23594<br>7.68	216	12	-.PPYTIVYFPVR.G<br>K.FEDGDLTLYQSNAILR.H	
14	heat shock protein 65	gi|51455	60903<br>5.91	871	16	R.TVIIEQSWGSPK.V<br>R.AAVEEGIVLGGGCALLR.C<br>K.ISSVQSIVPALEIANAHR.K<br>R.KPLVIIAEDVDGEALSTLVLNR.L<br>K.LVQDVANNTNEEAGDGTTTATVLAR.S	BC>BT<br>1.14<br>MC<BC<br>1.18<br>MC=BT
	chaperonin subunit 8 (theta), isoform CRA_b	gi|148665922	54021<br>5.19	96	4	R.FAEAFEAIPR.-<br>-.LFVTNDAATILR.F	
15	Sui1 homolog	gi|4868333	12668<br>6.04	208	26	K.TLTTVQGIADDYDK.K<br>K.GDDLLPAGTEDYIHIR.I	BC>BT<br>1.23

	serine (or cysteine) peptidase inhibitor, clade H, member 1, isoform CRA_a	gi|148684430	44954<br>9.01	94	2	R.DNQSGSLLFIGR.L	MC=BC<br>MC>BT<br>1.38
16	Stathmin	gi|9789995	17264<br>5.76	208	22	K.DKHVEEVR.K<br>K.DKHVEEVRK.N<br>K.ESVPDFPLSPPK.K<br>R.ASGQAFELILSPR.S	BC>BT<br>1.20<br>MC>BC<br>1.56<br>MC>BT<br>1.87
18	calcyclin binding protein	gi|3142331	28301<br>7.78	175	17	K.TDTVIILCR.K<br>K.ISNYGWDQSDK.F<br>K.IYITLTGVHQVPTENVQVHFTER.S	BC>BT<br>1.16<br>MC<BC<br>1.21<br>MC=BT
	eukaryotic translation initiation factor 4H	gi|15808988	27324<br>6.67	110	5	K.EALTYDGALLGDR.S	
19	phosphoglycerate kinase	gi|202423	44508<br>7.53	658	22	K.LGDVYVNDAFGTAHR.A<br>K.ALESPERPFLAILGGAK.V<br>K.ITLPVDFVTADKFDENAK.T<br>K.QIVWNGPVGVFEWEAFAR.G<br>K.QIVWNGPVGVFEWEAFAR.G<br>K.DCVGPEVENACANPAAGTVILLENLR.F	BC<BT<br>1.28<br>MC<BC<br>2.21<br>MC<BT<br>2.83
20	Prohibitin	gi|6679299	29802<br>5.57	265	16	R.FDAGELITQR.E<br>K.DLQNVNITLR.I<br>R.IYTSIGEDYDER.V<br>R.KLEAAEDIAYQLSR.S	BC<BT<br>1.13<br>MC=BC<br>MC=BT

	glyoxalase domain-containing protein 4	gi|255003777	33296<br>5.28	110	11	K.SDEWFATR.N<br>R.FQTVHFFR.D<br>K.LELQGIQGAVDHAAAFGR.I	
22	translationally-controlled tumor protein	gi|6678437	19450<br>4.76	146	13	K.GKLEEQKPER.V<br>R.DLISHDELFSDIYK.I	BC>BT<br>1.26<br>MC>BC<br>1.39<br>MC>BT<br>1.75
	prostaglandin E synthase 3	gi|9790017	18709<br>4.36	140	18	R.SILCCLR.K<br>R.KGESGQSWPR.L<br>K.LTFSCLGGSDNFK.H	
	eukaryotic translation initiation factor 3 subunit K isoform 1	gi|21312044	25070<br>4.81	87	5	R.YNPENLATLER.Y	
23	histidine triad nucleotide-binding protein 1	gi|33468857	13768<br>6.36	533	71	K.IIFEDDR.C<br>K.AQVAQPGGDTIFGK.I<br>R.CLAFHDISPQAPTHFLVIPK.K<br>R.MVVNEGADGGQSVYHIHLHVLGGR.Q<br>R.MVVNEGADGGQSVYHIHLHVLGGR.Q<br>K.KHISQISVADDDDESLLGHLMIVGK.K<br>K.KHISQISVADDDDESLLGHLMIVGK.K	BC=BT<br>MC<BC<br>1.68<br>MC<BT<br>1.50
24	protein disulfide-isomerase A3 precursor	gi|112293264	56643<br>5.88	690	20	K.QAGPASVPLR.T<br>K.DASVVGFFR.D<br>K.FVMQEEFSR.D<br>R.LAPEYEAAATR.L<br>R.FAHTNIESLVK.E<br>R.ELNDFISYLQR.E<br>K.EYDDNGEGITIFRPLHLANK.F<br>K.TFSHELSDFGLESTTGEVPVVAIR.T	BC=BT<br>MC<BC<br>1.36<br>MC<BT<br>1.31
25	fatty acid-binding protein, epidermal isoform 1	gi|6754450	15127<br>6.14	320	32	K.ELGVGLALR.K<br>K.FDETTADGR.K<br>R.LMESHGFEEYMK.E<br>R.LMESHGFEEYMK.E<br>K.MIVECVMNNATCTR.V<br>K.MIVECVMNNATCTR.V	BC=BT<br>MC<BC<br>1.35<br>MC<BT<br>1.36

26	10 kDa heat shock protein, mitochondrial	gi|6680309	10956<br>7.93	330	43	K.FLPLFDR.V<br>R.KFLPLFDR.V<br>K.VLLPEYGGTK.V<br>K.VLQATVVAVGSGGK.G<br>K.VVLDDKDYFLFR.D	BC=BT<br>MC<BC<br>1.57<br>MC<BT<br>1.53
27	Enolase 1, (alpha)	gi|59808815	47128<br>6.16	667	23	R.IGAEVYHNLK.N<br>R.GNPTVEVDLYTAK.G<br>R.YITPDQLADLYK.S<br>K.LAQSNGWGVMVSHR.S<br>R.AAVPSGASTGIYEALELR.D<br>K.LAMQEFMILPVGASSFR.E<br>K.AGYTDQVVIGMDVAASEFYR.S	BC=BT<br>MC<BC<br>1.33<br>MC=BT
28	cytochrome b5	gi|13385268	15232<br>4.96	426	41	K.STWVILHHK.V<br>K.TYIIGELHPDDR.S<br>K.FLEEHPGGEEVLR.E<br>R.EQAGGDATENFEDVGHSTDAR.E	BC=BT<br>MC<BC<br>5.28<br>MC<BT<br>5.03
29	glyceraldehyde-3-phosphate dehydrogenase	gi|6679937	35787<br>8.44	442	19	R.VPTPNVSVVDLTCR.L<br>K.LVINGKPITIFQER.D<br>K.LISWYDNEYGYSNR.V<br>K.VIHDNFGIVEGLMTTVHAITATQK.T	BC=BT<br>MC<BC<br>1.21<br>MC<BT<br>1.29
	L-lactate dehydrogenase A chain isoform 1	gi|6754524	36475<br>7.62	151	9	K.LVIITAGAR.Q<br>K.VTLTPEEEAR.L<br>R.VIGSGCNLDSAR.F	
30	protein disulfide-isomerase A3 precursor	gi|112293264	56643<br>5.88	690	20	K.QAGPASVPLR.T<br>K.DASVVGFFR.D<br>K.FVMQEEFSR.D<br>R.LAPEYEAAATR.L<br>R.FAHTNIESLVK.E<br>R.ELNDFISYLQR.E<br>K.EYDDNGEGITIFRPLHLANK.F<br>K.TFSHELSDFGLESTTGEVPVVAIR.T	BC=BT<br>MC<BC<br>1.29<br>MC=BT
	alpha-fetoprotein, partial	gi|191765	47195<br>5.47	212	9	K.APQVSTPTLVEAAR.N<br>K.LGEYGFQNAILVR.Y<br>K.DVFLGTFLYEYSR.R	

31	Elongation factor 1-delta	gi|13124192	31274<br>4.91	272	17	R.ITSLEVENQNLR.G<br>R.FYEQMNGPVTSGSR.Q<br>K.SLAGSSGPGASSGPGGDHSELIVR.I	BC=BT<br>MC>BC<br>1.19<br>MC=BT
32	phosphoglycerate kinase	gi|202423	44508<br>7.53	658	22	K.LGDVYVNDAFGTAHR.A<br>K.ALESPERPFLAILGGAK.V<br>K.ITLPVDFVTADKFDENAK.T<br>K.QIVWNGPVGVFEWEAFAR.G<br>K.QIVWNGPVGVFEWEAFAR.G<br>K.DCVGPEVENACANPAAGTVILLENLR.F	BC=BT<br>MC<BC<br>1.80<br>MC<BT<br>1.94
	long-chain acyl-CoA dehydrogenase	gi|726095	48052<br>8.34	154	8	R.IFSSEHDIFR.E<br>K.AQDTAELFFEDVR.L<br>K.GFYYLMQELPQER.L	
	heparin binding protein 44	gi|220434	42058<br>7.49	104	6	K.LIHNLNVILAR.Y<br>K.IQEYNVLLDTLSR.A	
33	beta-galactoside binding protein	gi|193442	14796<br>5.32	341	33	K.LPDGHEFK.F<br>K.EDGTWGTEHR.E<br>K.DSNNLCLHFNPR.F<br>R.FNAHGDANTIVCNTK.E	BC=BT<br>MC<BC<br>1.99<br>MC<BT<br>1.97
35	10 kDa heat shock protein, mitochondrial	gi|6680309	10956<br>7.93	330	43	K.FLPLFDR.V<br>R.KFLPLFDR.V<br>K.VLLPEYGGTK.V<br>K.VLQATVVAVGSGGK.G<br>K.VVLDDKDYFLFR.D	BC=BT<br>MC<BC<br>1.56<br>MC<BT<br>1.69
	macrophage migration inhibitory fator	gi|6754696	12496<br>6.79	166	17	K.LLCGLLSDR.L<br>M.PMFIVNTNVPR.A<br>M.PMFIVNTNVPR.A	
36	D-dopachrome decarboxylase	gi|6753618	13069<br>6.09	68	11	M.PFVELETNLPASR.I	BC=BT<br>MC<BC<br>2.05<br>MC=BT

38	Stress-induced phosphoprotein 1	gi|13277819	62528<br>6.40	201	5	K.AAALEFLNR.F<br>K.LMDVGLIAIR.-<br>R.KAAALEFLNR.F<br>K.LDPQNHVLYSNR.S	BC=BT<br>MC>BC<br>1.30<br>MC>BT<br>1.46
	Matricin	gi|347839	60450<br>6.23	73	4	R.AVAQALEVIPR.T<br>K.TAVETAVLLLR.I	
40	guanosine diphosphate (GDP) dissociation inhibitor 2	gi|148700276	52911<br>8.66	98	4	K.MLLFTEVTR.Y<br>K.DLGTDSQIFISR.A	BC=BT<br>MC<BC<br>1.19<br>MC=BT
41	glyceraldehyde-3-phosphate dehydrogenase	gi|6679937	35787<br>8.44	442	19	R.VPTPNVSVVDLTCR.L<br>K.LVINGKPITIFQER.D<br>K.LISWYDNEYGYSNR.V<br>K.VIHDNFGIVEGLMTTVHAITATQK.T	BC=BT<br>MC<BC<br>1.27<br>MC=BT
43	cytochrome b5	gi|13385268	15232<br>4.96	426	41	K.STWVILHHK.V<br>K.TYIIGELHPDDR.S<br>K.FLEEHPGGEEVLR.E<br>R.EQAGGDATENFEDVGHSTDAR.E	BC=BT<br>MC>BC<br>1.40<br>MC>BT<br>1.72
	eukaryotic translation initiation factor 5a	gi|148680531	18356<br>5.08	62	7	K.VHLVGIDIFTGK.K	
44	phosphoglycerate kinase	gi|202423	44508<br>7.53	658	22	K.LGDVYVNDAFGTAHR.A<br>K.ALESPERPFLAILGGAK.V<br>K.ITLPVDFVTADKFDENAK.T<br>K.QIVWNGPVGVFEWEAFAR.G<br>K.QIVWNGPVGVFEWEAFAR.G<br>K.DCVGPEVENACANPAAGTVILLENLR.F	BC=BT<br>MC<BC<br>1.73<br>MC<BT<br>2.09

45	macrophage migration inhibitory fator	gi|6754696	12496<br>6.79	166	17	K.LLCGLLSDR.L<br>M.PMFIVNTNVPR.A<br>M.PMFIVNTNVPR.A	BC=BT<br>MC<BC<br>1.50<br>MC<BT<br>1.68
46	heat shock protein 70 cognate	gi|309319	70793<br>5.37	646	14	K.FELTGIPPAPR.G<br>K.VEIIANDQGNR.T<br>R.FEELNADLFR.G<br>R.RFDDAVVQSDMK.H<br>R.TTPSYVAFTDTER.L<br>K.STAGDTHLGGEDFDNR.M<br>K.TVTNAVVTVPAYFNDSQR.Q	BC=BT<br>MC>BC<br>2.05<br>MC=BT
	glyceraldehyde-3-phosphate dehydrogenase	gi|6679937	35787<br>8.44	442	19	R.VPTPNVSVVDLTCR.L<br>K.LVINGKPITIFQER.D<br>K.LISWYDNEYGYSNR.V<br>K.VIHDNFGIVEGLMTTVHAITATQK.T	
48	protein disulfide-isomerase A3 precursor	gi|112293264	56643<br>5.88	690	20	K.QAGPASVPLR.T<br>K.DASVVGFFR.D<br>K.FVMQEEFSR.D<br>R.LAPEYEAAATR.L<br>R.FAHTNIESLVK.E<br>R.ELNDFISYLQR.E<br>K.EYDDNGEGITIFRPLHLANK.F<br>K.TFSHELSDFGLESTTGEVPVVAIR.T	BC=BT<br>MC<BC<br>1.35<br>MC<BT<br>1.32
53	nucleophosmin isoform 1	gi|6679108	32540<br>4.62	417	19	K.DLKPSTPR.S<br>K.VDNDENEHQLSLR.T<br>R.MTDQEAIQDLWQWR.K<br>R.MTDQEAIQDLWQWR.K<br>K.MSVQPTVSLGGFEITPPVVLR.L<br>K.MSVQPTVSLGGFEITPPVVLR.L	BC=BT<br>MC<BC<br>1.47<br>MC=BT
56	phosphoglycerate kinase	gi|202423	44508<br>7.53	658	22	K.LGDVYVNDAFGTAHR.A<br>K.ALESPERPFLAILGGAK.V<br>K.ITLPVDFVTADKFDENAK.T<br>K.QIVWNGPVGVFEWEAFAR.G<br>K.QIVWNGPVGVFEWEAFAR.G<br>K.DCVGPEVENACANPAAGTVILLENLR.F	BC=BT<br>MC<BC<br>1.80<br>MC<BT<br>2.22

58	peptidyl-prolyl cis-trans isomerase A	gi|6679439	17960<br>7.74	394	37	R.VSFELFADK.V<br>K.FEDENFILK.H<br>K.EGMNIVEAMER.F<br>R.IIPGFMCQGGDFTR.H<br>R.IIPGFMCQGGDFTR.H<br>M.VNPTVFFDITADDEPLGR.V	BC=BT<br>MC<BC<br>1.26<br>MC=BT
59	Stress-induced phosphoprotein 1	gi|13277819	62528<br>6.40	201	5	K.AAALEFLNR.F<br>K.LMDVGLIAIR.-<br>R.KAAALEFLNR.F<br>K.LDPQNHVLYSNR.S	BC=BT<br>MC>BC<br>1.40<br>MC>BT<br>1.47
61	protein disulfide isomerase-related protein 5	gi|1710248	46170<br>4.95	107	7	K.GSFSEQGINEFLR.E<br>-.LYSSSDDVIELTPSNFNR.E	BC=BT<br>MC>BC<br>1.47<br>MC>BT<br>1.59
62	glyceraldehyde-3-phosphate dehydrogenase	gi|6679937	35787<br>8.44	442	19	R.VPTPNVSVVDLTCR.L<br>K.LVINGKPITIFQER.D<br>K.LISWYDNEYGYSNR.V<br>K.VIHDNFGIVEGLMTTVHAITATQK.T	BC=BT<br>MC<BC<br>1.22<br>MC<BT<br>1.30
	peroxiredoxin-1	gi|6754976	22162<br>8.26	218	20	K.IGYPAPNFK.A<br>R.TIAQDYGVLK.A<br>R.LVQAFQFTDK.H<br>R.QITINDLPVGR.S<br>R.QITINDLPVGR.S	
	peroxiredoxin-4 precursor	gi|7948999	31033<br>6.67	82	4	R.QITLNDLPVGR.S<br>R.QITLNDLPVGR.S	
63	glyceraldehyde-3-phosphate dehydrogenase	gi|6679937	35787<br>8.44	442	19	R.VPTPNVSVVDLTCR.L<br>K.LVINGKPITIFQER.D<br>K.LISWYDNEYGYSNR.V<br>K.VIHDNFGIVEGLMTTVHAITATQK.T	BC=BT<br>MC<BC<br>1.26<br>MC<BT<br>1.25
64	glutathione S-transferase P 1	gi|10092608	23594<br>7.68	216	12	-.PPYTIVYFPVR.G<br>K.FEDGDLTLYQSNAILR.H	BC=BT<br>MC>BC

	thioredoxin-dependent peroxide reductase, mitochondrial precursor	gi|6680690	28109<br>7.15	173	9	K.HLSVNDLPVGR.S<br>R.DYGVLLESAGIALR.G	1.30<br>MC>BT<br>1.35
	PEST proteolytic signal-containing nuclear protein	gi|71480098	18951<br>6.86	83	6	K.FGFAIGSQTAR.K	
67	Stress-induced phosphoprotein 1	gi|13277819	62528<br>6.40	201	5	K.AAALEFLNR.F<br>K.LMDVGLIAIR.-<br>R.KAAALEFLNR.F<br>K.LDPQNHVLYSNR.S	BC=BT<br>MC>BC<br>1.41<br>MC>BT<br>1.45
68	Chain E, Cytochrome C Oxidase At The Fully Oxidized State	gi|1942990	12428<br>5.01	178	21	R.LNDFASAVR.I<br>-.SHGSHETDEEFDAR.W	BC=BT<br>MC<BC<br>1.59<br>MC=BT
70	aldolase C	gi|229506	38993<br>9.06	195	14	K.ELSDIAHR.I<br>R.QLLLTADDR.V<br>K.ADDGRPFPQVIK.S<br>K.GVVPLAGTDGETTTQGLDGLSER.C	BC=BT<br>MC<BC<br>1.42<br>MC<BT<br>1.67
71	phosphoglycerate kinase	gi|202423	44508<br>7.53	658	22	K.LGDVYVNDAFGTAHR.A<br>K.ALESPERPFLAILGGAK.V<br>K.ITLPVDFVTADKFDENAK.T<br>K.QIVWNGPVGVFEWEAFAR.G<br>K.QIVWNGPVGVFEWEAFAR.G<br>K.DCVGPEVENACANPAAGTVILLENLR.F	BC=BT<br>MC<BC<br>1.41<br>MC=BT
76	heat shock protein 70 cognate	gi|309319	70793<br>5.37	646	14	K.FELTGIPPAPR.G<br>K.VEIIANDQGNR.T<br>R.FEELNADLFR.G<br>R.RFDDAVVQSDMK.H<br>R.TTPSYVAFTDTER.L<br>K.STAGDTHLGGEDFDNR.M<br>K.TVTNAVVTVPAYFNDSQR.Q	BC=BT<br>MC>BC<br>1.69<br>MC>BT<br>1.98
	mitochondrial stress-70 protein	gi|407341	73483<br>5.91	313	8	K.VQQTVQDLFGR.A<br>K.SDIGEVILVGGMTR.M<br>K.LLGQFTLIGIPPAPR.G<br>K.NAVITVPAYFNDSQR.Q	

77	Prostaglandina E synthase 3	gi|9790017	18709<br>4.36	140	18	R.SILCCLR.K<br>R.KGESGQSWPR.L<br>K.LTFSCLGGSDNFK.H	BC=BT<br>MC>BC<br>1.33<br>MC>BT<br>1.63
79	pyruvate kinase M	gi|551295	57824<br>7.58	687	24	K.GDYPLEAVR.M<br>R.LDIDSAPITAR.N<br>R.NTGIICTIGPASR.S<br>K.IYVDDGLISLQVK.E<br>K.DAVLNAWAEDVDLR.V<br>R.FDEILEASDGIMVAR.G<br>R.LNFSHGTHEYHAETIK.N<br>R.EAEAAIYHLQLFEELR.R<br>R.EATESFASDPILYRPVAVALDTK.G	BC=BT<br>MC>BC<br>1.19<br>MC=BT
80	nucleophosmin isoform 1	gi|6679108	32540<br>4.62	417	19	K.DLKPSTPR.S<br>K.VDNDENEHQLSLR.T<br>R.MTDQEAIQDLWQWR.K<br>R.MTDQEAIQDLWQWR.K<br>K.MSVQPTVSLGGFEITPPVVLR.L<br>K.MSVQPTVSLGGFEITPPVVLR.L	BC=BT<br>MC<BC<br>1.35<br>MC=BT
	calreticulin precursor	gi|6680836	47965<br>4.33	249	9	K.EQFLDGDAWTNR.W<br>K.HEQNIDCGGGYVK.L<br>K.IKDPDAAKPEDWDER.A	
81	mCG13235	gi|148684097	15406<br>9.82	188	25	K.EGLPPDQQR.L<br>K.ESTLHLVLR.L<br>K.TITLEVEPSDTIENVK.A	BC=BT<br>MC>BC<br>1.64<br>MC=BT
	polyubiquitin, partial	gi|1675359	4605<br>4.72	118	39	K.TITLEVEPSDTIENVK.A<br>K.TITLEVEPDDTIENVK.A	
	NEDD8 precursor	gi|6679034	8967<br>6.59	106	34	K.ILGGSVLHLVLALR.G<br>K.EIEIDIEPTDKVER.I	
83	alpha-fetoprotein, partial	gi|191765	47195<br>5.47	212	9	K.APQVSTPTLVEAAR.N<br>K.LGEYGFQNAILVR.Y<br>K.DVFLGTFLYEYSR.R	BC=BT<br>MC>BC<br>1.38<br>MC>BT<br>1.26

84	gamma-actin	gi|809561	40992<br>5.56	691	25	K.AGFAGDDAPR.A<br>R.GYSFTTTAER.E<br>R.AVFPSIVGRPR.H<br>K.QEYDESGPSIVHR.K<br>K.IWHHTFYNELR.V<br>K.SYELPDGQVITIGNER.F<br>K.DLYANTVLSGGTTMYPGIADR.M<br>K.DLYANTVLSGGTTMYPGIADR.M	BC=BT<br>MC<BC<br>1.05<br>MC=BT
	put. beta-actin (aa 27-375)	gi|49868	39161<br>5.78	629	22	R.AVFPSIVGR.S<br>R.GYSFTTTAER.E<br>K.QEYDESGPSIVHR.K<br>K.IWHHTFYNELR.V<br>K.SYELPDGQVITIGNER.F<br>K.DLYANTVLSGGTTMYPGIADR.M<br>K.DLYANTVLSGGTTMYPGIADR.M	
	Eukaryotic translation initiation factor 3, subunit F	gi|47682703	37857<br>5.33	175	16	K.YAYYDTER.I<br>R.VIGLSSDLQQVGGASAR.I<br>R.LHPVILASIVDSYER.R<br>R.EAPNPIHLTVDTGLQHGR.M	
85	Vimentin	gi|55408	53689<br>5.06	455	17	R.DNLAEDIMR.L<br>K.FADLSEAANR.N<br>K.VELQELNDR.F<br>R.LGDLYEEEMR.D<br>K.MALDIEIATYR.K<br>R.ISLPLPTFSSLNLR.E<br>R.EMEENFALEAANYQDTIGR.L	BC=BT<br>MC>BC<br>1.30<br>MC=BT
87	glyceraldehyde-3-phosphate dehydrogenase	gi|6679937	35787<br>8.44	442	19	R.VPTPNVSVVDLTCR.L<br>K.LVINGKPITIFQER.D<br>K.LISWYDNEYGYSNR.V<br>K.VIHDNFGIVEGLMTTVHAITATQK.T	BC=BT<br>MC=BC<br>MC<BT<br>1.20
88	annexin A5	gi|6753060	35730<br>4.83	267	18	K.FITIFGTR.S<br>R.SEIDLFNIR.K<br>R.GTVTDFPGFDGR.A<br>K.GLGTDEDSILNLLTSR.S<br>K.YMTISGFQIEETIDR.E	BC=BT<br>MC=BC<br>MC>BT<br>1.33

91	glyceraldehyde-3-phosphate dehydrogenase	gi|6679937	35787<br>8.44	442	19	R.VPTPNVSVVDLTCR.L<br>K.LVINGKPITIFQER.D<br>K.LISWYDNEYGYSNR.V<br>K.VIHDNFGIVEGLMTTVHAITATQK.T	BC=BT<br>MC=BC<br>MC<BT<br>1.45
	aldolase C	gi|229506	38993<br>9.06	195	14	K.ELSDIAHR.I<br>R.QLLLTADDR.V<br>K.ADDGRPFPQVIK.S<br>K.GVVPLAGTDGETTTQGLDGLSER.C	
92	glyceraldehyde-3-phosphate dehydrogenase	gi|6679937	35787<br>8.44	442	19	R.VPTPNVSVVDLTCR.L<br>K.LVINGKPITIFQER.D<br>K.LISWYDNEYGYSNR.V<br>K.VIHDNFGIVEGLMTTVHAITATQK.T	BC=BT<br>MC=BC<br>MC<BT<br>1.41
	alcohol dehydrogenase [NADP(+)]	gi|10946870	36564<br>6.90	75	6	K.ALGLSNFNSR.Q<br>K.HHPEDVEPALR.K	
93	beta-galactoside binding protein	gi|193442	14796<br>5.32	341	33	K.LPDGHEFK.F<br>K.EDGTWGTEHR.E<br>K.DSNNLCLHFNPR.F<br>R.FNAHGDANTIVCNTK.E	BC=BT<br>MC=BC<br>MC<BT<br>1.40
94	T-complex protein 1 subunit zeta	gi|6753324	57968<br>6.63	95	2	K.QADLYISEGLHPR.I<br>K.QADLYISEGLHPR.I	BC=BT<br>MC=BC<br>MC>BT<br>1.23
95	phosphoglycerate kinase	gi|202423	44508<br>7.53	658	22	K.LGDVYVNDAFGTAHR.A<br>K.ALESPERPFLAILGGAK.V<br>K.ITLPVDFVTADKFDENAK.T<br>K.QIVWNGPVGVFEWEAFAR.G<br>K.QIVWNGPVGVFEWEAFAR.G<br>K.DCVGPEVENACANPAAGTVILLENLR.F	BC=BT<br>MC=BC<br>MC<BT<br>1.59
96	1,2-dihydroxy-3-keto-5-methylthiopentene dioxygenase	gi|19527270	21510<br>5.31	92	6	R.YILEGSGYFDVR.D	BC=BT<br>MC=BC<br>MC>BT<br>1.21
	39S ribosomal protein L12, mitochondrial precursor	gi|22164792	21695<br>9.34	78	5	K.NYVQGINLVQAK.K	

98	heat shock protein 70 cognate	gi|309319	70793<br>5.37	646	14	K.FELTGIPPAPR.G<br>K.VEIIANDQGNR.T<br>R.FEELNADLFR.G<br>R.RFDDAVVQSDMK.H<br>R.TTPSYVAFTDTER.L<br>K.STAGDTHLGGEDFDNR.M<br>K.TVTNAVVTVPAYFNDSQR.Q	BC=BT<br>MC=BC<br>MC>BT<br>1.70
	mitochondrial stress-70 protein	gi|407341	73483<br>5.91	313	8	K.VQQTVQDLFGR.A<br>K.SDIGEVILVGGMTR.M<br>K.LLGQFTLIGIPPAPR.G<br>K.NAVITVPAYFNDSQR.Q	
100	vacuolar protein-sorting-associated protein 25 isoform 2	gi|25092662	20735<br>5.97	192	17	K.SSFLIMWR.R<br>K.LIYQWVSR.S<br>R.FPPFFTLQPNVDTR.Q	BC=BT<br>MC=BC<br>MC<BT<br>1.28
101	pyruvate kinase M	gi|551295	57824<br>7.58	687	24	K.GDYPLEAVR.M<br>R.LDIDSAPITAR.N<br>R.NTGIICTIGPASR.S<br>K.IYVDDGLISLQVK.E<br>K.DAVLNAWAEDVDLR.V<br>R.FDEILEASDGIMVAR.G<br>R.LNFSHGTHEYHAETIK.N<br>R.EAEAAIYHLQLFEELR.R<br>R.EATESFASDPILYRPVAVALDTK.G	BC=BT<br>MC=BC<br>MC<BT<br>1.35
	tubulin beta-5 chain	gi|7106439	49639<br>4.78	90	6	R.FPGQLNADLR.K<br>K.GHYTEGAELVDSVLDVVR.K	
102	peptidyl-prolyl cis-trans isomerase A	gi|6679439	17960<br>7.74	394	37	R.VSFELFADK.V<br>K.FEDENFILK.H<br>K.EGMNIVEAMER.F<br>R.IIPGFMCQGGDFTR.H<br>R.IIPGFMCQGGDFTR.H<br>M.VNPTVFFDITADDEPLGR.V	BC=BT<br>MC=BC<br>MC<BT<br>1.13
103	thioredoxin-dependent peroxide reductase, mitochondrial precursor	gi|6680690	28109<br>7.15	173	9	K.HLSVNDLPVGR.S<br>R.DYGVLLESAGIALR.G	BC=BT<br>MC=BC<br>MC>BT

	trafficking protein particle complex subunit 4	gi|11140825	24370<br>5.83	135	17	K.FVVLADPR.Q<br>K.AGGLIYQWDSYSPR.A<br>K.EVLEYLGNPANYPVSIR.F	1.54
	ER membrane protein complex subunit 8	gi|6754870	23333<br>5.72	106	7	R.SYETLVDFDNHLDDIR.N	
104	beta-galactoside binding protein	gi|193442	14796<br>5.32	341	33	K.LPDGHEFK.F<br>K.EDGTWGTEHR.E<br>K.DSNNLCLHFNPR.F<br>R.FNAHGDANTIVCNTK.E	BC=BT<br>MC=BC<br>MC<BT<br>1.62
105	glyceraldehyde-3-phosphate dehydrogenase	gi|6679937	35787<br>8.44	442	19	R.VPTPNVSVVDLTCR.L<br>K.LVINGKPITIFQER.D<br>K.LISWYDNEYGYSNR.V<br>K.VIHDNFGIVEGLMTTVHAITATQK.T	BC=BT<br>MC=BC<br>MC<BT<br>1.46
106	glyceraldehyde-3-phosphate dehydrogenase	gi|6679937	35787<br>8.44	442	19	R.VPTPNVSVVDLTCR.L<br>K.LVINGKPITIFQER.D<br>K.LISWYDNEYGYSNR.V<br>K.VIHDNFGIVEGLMTTVHAITATQK.T	BC=BT<br>MC=BC<br>MC<BT<br>1.37
107	glyceraldehyde-3-phosphate dehydrogenase	gi|6679937	35787<br>8.44	442	19	R.VPTPNVSVVDLTCR.L<br>K.LVINGKPITIFQER.D<br>K.LISWYDNEYGYSNR.V<br>K.VIHDNFGIVEGLMTTVHAITATQK.T	BC=BT<br>MC=BC<br>MC<BT<br>1.18
108	Destrin	gi|9790219	18509<br>8.14	95	7	K.HEYQANGPEDLNR.T	BC=BT<br>MC=BC<br>MC>BT<br>1.30
110	14-3-3 protein epsilon	gi|13928824	29103<br>4.55	360	20	K.DSTLIMQLLR.D<br>K.DSTLIMQLLR.D<br>R.YLAEFATGNDR.K<br>R.YLAEFATGNDRK.E<br>K.VAGMDVELTVEER.N<br>K.AASDIAMTELPPTHPIR.L<br>K.AASDIAMTELPPTHPIR.L	BC=BT<br>MC=BC<br>MC<BT<br>1.16

113	phosphoglycerate mutase 1	gi|114326546	28814<br>6.67	824	54	R.VLIAAHGNSLR.G<br>R.HGESAWNLENR.F<br>R.ALPFWNEEIVPQIK.E<br>R.DAGYEFDICFTSVQK.R<br>R.FSGWYDADLSPAGHEEAK.R<br>K.NLKPIKPMQFLGDEETVR.K<br>K.NLKPIKPMQFLGDEETVR.K<br>R.SYDVPPPPMEPDHPFYSNISK.D	BC=BT<br>MC=BC<br>MC<BT<br>1.32
	calcyclin binding protein	gi|3142331	28301<br>7.78	175	18	K.TDTVIILCR.K<br>K.ISNYGWDQSDK.F<br>K.IYITLTGVHQVPTENVQVHFTER.S	
114	Enolase 1, (alpha)	gi|59808815	47128<br>6.16	667	23	R.IGAEVYHNLK.N<br>R.GNPTVEVDLYTAK.G<br>R.YITPDQLADLYK.S<br>K.LAQSNGWGVMVSHR.S<br>R.AAVPSGASTGIYEALELR.D<br>K.LAMQEFMILPVGASSFR.E<br>K.AGYTDQVVIGMDVAASEFYR.S	BC=BT<br>MC=BC<br>MC<BT<br>1.29
116	alpha-fetoprotein, partial	gi|191765	47195<br>5.47	212	9	K.APQVSTPTLVEAAR.N<br>K.LGEYGFQNAILVR.Y<br>K.DVFLGTFLYEYSR.R	BC=BT<br>MC=BC<br>MC<BT<br>1.40
	F-actin-capping protein subunit beta isoform a	gi|83649737	31326<br>5.47	84	5	K.LEVEANNAFDQYR.D<br>R.KLEVEANNAFDQYR.D	
117	glyceraldehyde-3-phosphate dehydrogenase	gi|6679937	35787<br>8.44	442	19	R.VPTPNVSVVDLTCR.L<br>K.LVINGKPITIFQER.D<br>K.LISWYDNEYGYSNR.V<br>K.VIHDNFGIVEGLMTTVHAITATQK.T	BC=BT<br>MC=BC<br>MC<BT<br>1.46
	macrophage migration inhibitory fator	gi|6754696	12496<br>6.79	166	17	K.LLCGLLSDR.L<br>M.PMFIVNTNVPR.A<br>M.PMFIVNTNVPR.A	

GI: GenInfo code; pI: isoeletric point; BC: B16-F10 control; BT: B16-F10 treated; MC: Melan-a control and MT: Melan-a treated.

Supplemental table 2: Functional categories of the identified proteins.

Functional category	Spot #
01. Metabolism	
1,2-dihydroxy-3-keto-5-methylthiopentene dioxygenase	96
Alcohol dehydrogenase [nadp(+)]	92
Aldolase c	70; 91
Alpha-fetoprotein, partial	30; 83; 116
Cytochrome b5	28; 43
D-dopachrome decarboxylase	36
Enolase 1, (alpha)	2; 27; 114
Fatty acid-binding protein, epidermal isoform 1	25
Glutathione s-transferase p 1	13; 64
Glyceraldehyde-3-phosphate dehydrogenase	13; 29; 41; 46; 62; 63; 87; 91; 92; 105; 106; 107; 117
L-lactate dehydrogenase a chain isoform 1	29
Long-chain acyl-coa dehydrogenase	32
Macrophage migration inhibitory fator	35; 45; 117
Phosphoglycerate kinase	1; 19; 32; 44; 56;71; 95
Phosphoglycerate mutase 1	5; 8; 113
Prostaglandin e synthase 3	22
Pyruvate kinase m	10; 79; 101
Triosephosphate isomerase	7
Tubulin beta-5 chain	101
02. Energy	
Aldolase c	70; 91
Enolase 1, (alpha)	2; 27; 114
L-lactate dehydrogenase a chain isoform 1	29
Long-chain acyl-coa dehydrogenase	32
Phosphoglycerate kinase	1; 19; 32; 44; 56;71; 95
Phosphoglycerate mutase 1	5; 8; 113
Pyruvate kinase m	10; 79; 101
Triosephosphate isomerase	7
10. Cell cycle and DNA processing	
Calreticulin precursor	80
Cofilin-1	9
Destrin	108
Heat shock protein 70 cognate	4; 11; 46; 76; 98
Prohibitin	20
Stathmin	16
Tubulin beta-5 chain	101
Vacuolar protein-sorting-associated protein 25 isoform 2	100
11. Transcription	
Nedd8 precursor	81
Vacuolar protein-sorting-associated protein 25 isoform 2	100
12. Protein synthesis	
39s ribosomal protein l12, mitochondrial precursor	96
Elongation factor 1-delta	31
Eukaryotic translation initiation factor 3 subunit k isoform 1	22
Eukaryotic translation initiation factor 3, subunit f	84
Eukaryotic translation initiation factor 4h	18
Eukaryotic translation initiation factor 5a	43
Sui1 homolog	15
14. Protein fate (folding, modification, destination)	
10 kda heat shock protein, mitochondrial	26; 35
14-3-3 protein épsilon	110

Calcyclin binding protein	18; 113
Calreticulin precursor	80
Heat shock protein 65	3; 14
Heat shock protein 70 cognate	4; 11; 46; 76; 98
Mitochondrial stress-70 protein	76; 98
Nedd8 precursor	81
Nucleophosmin isoform 1	12; 53; 80
Phosphoglycerate kinase	1; 19; 32; 44; 56;71; 95
Protein disulfide-isomerase a3 precursor	24; 30; 48
T-complex protein 1 subunit zeta	94
Tubulin beta-5 chain	101
16. Protein with binding function or cofactor requirement (structural or catalytic)	
1,2-dihydroxy-3-keto-5-methylthiopentene dioxygenase	96
10 kda heat shock protein, mitochondrial	26; 35
14-3-3 protein épsilon	110
39s ribosomal protein l12, mitochondrial precursor	96
Aldolase c	70; 91
Alpha-fetoprotein, partial	30; 83; 116
Annexin a5	88
Beta-galactoside binding protein	33; 93; 104
Calreticulin precursor	80
Cofilin-1	9
Cytochrome b5	28; 43
Destrin	108
Elongation factor 1-delta	31
Enolase 1, (alpha)	2; 27; 114
Eukaryotic translation initiation factor 4h	18
F-actin-capping protein subunit beta isoform a	116
Fatty acid-binding protein, epidermal isoform 1	25
Guanosine diphosphate (gdp) dissociation inhibitor 2	40
Heat shock protein 70 cognate	4; 11; 46; 76; 98
L-lactate dehydrogenase a chain isoform 1	29
Long-chain acyl-coa dehydrogenase	32
Mitochondrial stress-70 protein	76; 98
Nedd8 precursor	81
Nucleophosmin isoform 1	12; 53; 80
Phosphoglycerate kinase	1; 19; 32; 44; 56;71; 95
Pyruvate kinase m	10; 79; 101
Stathmin	16
Stress-induced phosphoprotein 1	38; 59; 67
T-complex protein 1 subunit zeta	94
Thioredoxin-dependent peroxide reductase, mitochondrial precursor	64; 103
Trafficking protein particle complex subunit 4	103
Translationally-controlled tumor protein	22
Tubulin beta-5 chain	101
Vimentin	85
18. Regulation of metabolism and protein function	
Elongation factor 1-delta	31
Guanosine diphosphate (gdp) dissociation inhibitor 2	40
Rho gdp dissociation inhibitor (gdi) alpha	11
20. Cellular transport, transport facilities and transport routes	
1-cys peroxiredoxin protein	7
Alpha-fetoprotein, partial	30; 83; 116
Cytochrome b5	28; 43
Fatty acid-binding protein, epidermal isoform 1	25
Long-chain acyl-coa dehydrogenase	32
Mitochondrial stress-70 protein	76; 98

Peroxiredoxin-1	62
Trafficking protein particle complex subunit 4	103
Tubulin beta-5 chain	101
Vacuolar protein-sorting-associated protein 25 isoform 2	100
30. Cellular communication/signal transduction mechanism	
Beta-galactoside binding protein	33; 93; 104
Rho gdp dissociation inhibitor (gdi) alpha	11
Stathmin	16
32. Cell rescue, defense and virulence	
1-cys peroxiredoxin protein	7
Glutathione s-transferase p 1	13; 64
Heat shock protein 70 cognate	4; 11; 46; 76; 98
Macrophage migration inhibitory fator	35; 45; 117
Mitochondrial stress-70 protein	76; 98
Nucleophosmin isoform 1	12; 53; 80
Peroxiredoxin-1	62
Peroxiredoxin-4 precursor	62
Thioredoxin-dependent peroxide reductase, mitochondrial precursor	64; 103
34. Interaction with the environment	
Cofilin-1	9
Destrin	108
Heat shock protein 70 cognate	4; 11; 46; 76; 98
Mitochondrial stress-70 protein	76; 98
Stathmin	16
36. Systemic interaction with the environment	
Annexin a5	88
Macrophage migration inhibitory fator	35; 45; 117
40. Cell fate	
Cofilin-1	9
Eukaryotic translation initiation factor 5a	43
F-actin-capping protein subunit beta isoform a	116
Macrophage migration inhibitory fator	35; 45; 117
Protein disulfide-isomerase a3 precursor	24; 30; 48
Translationally-controlled tumor protein	22
41. Development (Systemic)	
Cofilin-1	9
Stathmin	16
Trafficking protein particle complex subunit 4	103
42. Biogenesis of cellular components	
Calreticulin precursor	80
Cofilin-1	9
Destrin	108
F-actin-capping protein subunit beta isoform a	116
Stathmin	16
Tubulin beta-5 chain	101
Vacuolar protein-sorting-associated protein 25 isoform 2	100
Vimentin	85
43. Cell type differentiation	
Stathmin	16
Trafficking protein particle complex subunit 4	103
45. Tissue differentiation	
Alpha-fetoprotein, partial	30; 83; 116
Stathmin	16
47. Organ differentiation	
Alpha-fetoprotein, partial	30; 83; 116
Cofilin-1	9
Stathmin	16
